# TREM2 gene expression associations with Alzheimer’s disease neuropathology are region-specific: implications for cortical versus subcortical microglia

**DOI:** 10.1007/s00401-023-02564-2

**Published:** 2023-03-25

**Authors:** Rebecca L. Winfree, Mabel Seto, Logan Dumitrescu, Vilas Menon, Philip De Jager, Yanling Wang, Julie Schneider, David A. Bennett, Angela L. Jefferson, Timothy J. Hohman

**Affiliations:** 1 Vanderbilt Memory and Alzheimer’s Center, Vanderbilt University Medical Center, 1207 17th Ave S, Nashville, TN 37212 USA; 2 Department of Neurology, Vanderbilt University Medical Center, Nashville, TN USA; 3 Vanderbilt Genetics Institute, Vanderbilt University Medical Center, Nashville, TN USA; 4 Pharmacology Department, Vanderbilt University Medical Center, Nashville, TN USA; 5 Department of Neurology, Columbia University Medical Center, New York, NY USA; 6 Department of Pathology, Rush University Medical Center, Chicago, IL USA; 7 Rush Alzheimer’s Disease Center, Rush University Medical Center, Chicago, IL USA

**Keywords:** Alzheimer’s disease, TREM2, Microglia, Amyloid-β

## Abstract

Previous post-mortem assessments of *TREM2* expression and its association with brain pathologies have been limited by sample size. This study sought to correlate region-specific *TREM2* mRNA expression with diverse neuropathological measures at autopsy using a large sample size (*N* = 945) of bulk RNA sequencing data from the Religious Orders Study and Rush Memory and Aging Project (ROS/MAP). *TREM2* gene expression of the dorsolateral prefrontal cortex, posterior cingulate cortex, and caudate nucleus was assessed with respect to core pathology of Alzheimer’s disease (amyloid-β, and tau), cerebrovascular pathology (cerebral infarcts, arteriolosclerosis, atherosclerosis, and cerebral amyloid angiopathy), microglial activation (proportion of activated microglia), and cognitive performance. We found that cortical *TREM2* levels were positively related to AD diagnosis, cognitive decline, and amyloid-β neuropathology but were not related to the proportion of activated microglia. In contrast, caudate *TREM2* levels were not related to AD pathology, cognition, or diagnosis, but were positively related to the proportion of activated microglia in the same region. Diagnosis-stratified results revealed caudate *TREM2* levels were inversely related to AD neuropathology and positively related to microglial activation and longitudinal cognitive performance in AD cases. These results highlight the notable changes in *TREM2* transcript abundance in AD and suggest that its pathological associations are brain-region-dependent.

**Supplementary Information:**

The online version contains supplementary material available at 10.1007/s00401-023-02564-2.

## Introduction

The discovery of increased Alzheimer’s disease (AD) risk in carriers of rare Triggering Receptor Expressed on Myeloid Cells-2 (*TREM2*) mutations from genome-wide association studies [24, 31] jump-started the interest in characterizing the potential roles of TREM2 protein, and more generally innate immune function, in the progression of the disease. Since then, dysregulation of *TREM2* expression has also been documented in AD and other neuroinflammatory diseases. TREM2 signaling has been tied to mechanisms of phenotypic switching of microglia, regulation of neuropathology, and cognition [29, 30, 63]. However, there has yet to be a comprehensive evaluation of regional brain *TREM2* gene expression with all three of these critical components of AD in the human brain. The following analyses expand upon our understanding of how post-mortem *TREM2* mRNA levels in brain relate to disease by investigating regional, neuropathological, and retrospective cognitive functioning associations.

In addition to rare mutations in *TREM2* associated with increased late-onset AD (LOAD) risk, dysregulation of TREM2 expression is found in the AD brain. *TREM2* mRNA and protein expression are upregulated in severe AD frontal cortex versus aged control [42]. Furthermore, mRNA expression of *TREM2* has been found to track with both protein levels in the cortex as well as with clinical progression of the disease [42]. However, *TREM2* expression changes in AD may be regionally dependent as contrasting reports of TREM2 protein expression changes in the hippocampus—a region vulnerable early-on to the accumulation of AD-related pathology—yielded differential findings depending on the study and form of TREM2 measured [16, 42, 61]. A lack of differential expression changes in the frontal cortex was found between individuals with normal cognition and mild AD, which is consistent with the hypothesis that upregulation of *TREM2* expression is a late event which reflects a response to the accumulation of disease pathology [42].

Additionally, from transgenic mouse models of AD, several important findings were gleaned concerning expression changes of *TREM2* with respect to disease pathology. For example, TREM2 protein is enriched in brain regions associated with high levels of amyloid-β accumulation in mice similar to humans [21]. Specifically, TREM2 was highly expressed in microglia in temporal cortex surrounding plaques. This observation supports the most characterized role of TREM2 in microglia regulating pathological Aβ development [34, 57, 64, 65]. It was since determined that plaque-associated microglia upregulate levels of TREM2 and accompanying signaling adapter DAP12, indicating an essential role of this pathway is regulating amyloid-β plaque deposition in the brain parenchyma [21].

Accompanying the response of TREM2 protein to plaque development is a transcriptional and phenotypic transition from a maintenance to an activated microglial cell. The proportion of activated microglia (PAM) has been found to be elevated in AD compared to age-matched non-AD subjects [17, 20]. This increase in glial activation in disease is thought to include a reduction of microglial cell arborization without a reduction in total microglial cell density [48]. Furthermore, PAM in cortical tissue at autopsy has been tied most closely to increased Aβ neuropathology, and to a lesser extent increased tau neuropathology, among ROS/MAP participants [20]. In the same ROS/MAP study, authors found that PAM in subcortical regions did not relate to classical AD neuropathology or cognitive decline such as in cortical regions. We wonder, given these previous findings, whether *TREM2* transcription may relate to PAM in post-mortem tissue and whether this association is dependent on the presence of classical AD neuropathology. Additionally, we investigate whether PAM corresponds to *TREM2* levels in subcortical areas and if this may reflect age- or disease-related cerebrovascular changes as these areas contain smaller blood vessels residing in deep brain areas which are particularly vulnerable to injury from inflammation, arteriosclerosis, and ischemic lesions [45].

*TREM2* expression changes are associated with several neurodegenerative and inflammatory diseases [14, 26, 28, 44, 59]. Its non-specific immunological functions, including but not limited to those of microglial cells, suggests *TREM2* dysregulation may be associated with post-mortem amyloid-β levels as well as other concomitant pathology related to underlying immune dysregulation. Strong evidence links chronic inflammation to the development of small vessel disease and dementia. Effects of immune activation on blood vessels, such as proinflammatory cytokine, complement, or reactive oxygen species release by myeloid cells (microglia, dendritic cells, and monocytes), may manifest as localized infarcts, diffuse white matter hyperintensities, or microbleeds, increasing blood–brain barrier permeability in old age and disease [50]. This oxidative and inflammatory stress may affect blood vessels themselves and surrounding neurovascular unit components that aid in maintaining their structural and metabolic integrity. Several studies link TREM2 to these important neurovascular unit mechanisms [5, 15, 43, 56, 58, 59]. For example, loss of TREM2 disrupted the response of endothelial cell gene networks to vascular endothelial growth factor, suggesting an impairment in cell–cell networks related to vascular homeostasis known to lead to dysfunction in small vessel disease [15, 43]. Furthermore, the *TREM2* variant rs6918289 has been associated with increased risk of atherosclerosis [22].

The present analyses investigated the relationship between *TREM2* bulk transcript levels with cognitive functioning, microglial activation, and AD-related neuropathology, including measures of cerebrovascular pathology. We evaluated expression in the dorsolateral prefrontal cortex (dlPFC), the posterior cingulate cortex (PCC), and the head of the caudate nucleus (CN) to investigate the neuropathological correlates of *TREM2* levels across multiple brain regions. We hypothesized that higher expression of *TREM2* mRNA at autopsy will correspond to increases in AD neuropathology, microglial activation, cerebrovascular injury, and worse cognitive performance.

## Methods

### Participants

Autopsy and cognitive data from the Religious Orders Study (ROS) and the Rush Memory and Aging Project (MAP), or ROS/MAP, were leveraged to conduct this study [6]. Data collection began in 1994 and 1997, respectively, contributing to rich longitudinal clinical-pathologic information concerning risk factors in aging and AD. ROS enrolls religious clergy members from across the United States, while MAP enrolls lay persons from northeastern regions of Illinois. Participants are older, free of known dementia at baseline, and are predominantly of non-Hispanic white ancestry (see cohort demographics in Table 1). Importantly, all participants consented to organ donation. A Rush University Medical Center Institutional Review Board approved each study, and guidelines for data sharing within Institutional Review Board protocols. All participants signed informed and repository consents, and an Anatomic Gift Act. Additionally, analyses were approved by the Vanderbilt University Medical Center IRB.

**Table 1:** Participant characteristics by brain region

Characteristic	dlPFC	PCC	CN
*N*	945	523	718
AD pathological diagnosis, no. (%)	574 (61)	306 (59)	442 (62)
AD clinical diagnosis, no. (%)	397 (42)	192 (37)	279 (39)
Other dementia clinical diagnosis, no. (%)	15 (2)	9 (2)	9 (1)
MCI clinical diagnosis, no. (%)	234 (25)	134 (26)	193 (27)
No cognitive impairment, no. (%)	299 (32)	188 (36)	237 (33)
*APOE*-ε4 carrier, no. (%)	241 (26)	131 (25)	191 (27)
Male, no. (%)	323 (34)	197 (38)	251 (35)
Non-Hispanic white, no. (%)	930 (98)	514 (98)	712 (99)
Age at death (years)	89.5 ± 6.6	89.4 ± 6.5	89.3 ± 6.5
Education (years)	16.4 ± 3.6	16.4 ± 3.5	16.3 ± 3.6
Global cognition	− 0.8 ± 1.1	− 0.7 ± 1.0	− 0.8 ± 1.1
Post-mortem interval (hours)	7.6 ± 4.3	7.0 ± 4.0	7.6 ± 4.4
CERAD “moderate” or “frequent”, no. (%)	619 (66)	327 (63)	474 (66)
Braak III-VI, no. (%)	786 (83)	427 (82)	599 (83)
^†^Thal 3–5, no. (%)	594/772 (77)	321/415 (77)	458/588 (78)

Values are presented as mean ± standard deviation, unless otherwise indicated

Consortium to Establish a Registry for Alzheimer’s disease (CERAD) protocol for neuritic amyloid plaque density scores: (“none”, “sparse”, “moderate”, or “frequent”). Braak staging for neurofibrillary tangle distribution and severity (from 0; least severe, to VI; most severe)

*AD* Alzheimer’s disease, *MCI* mild cognitive impairment, *APOE-ε4* apolipoprotein E epsilon 4, *dlPFC* Dorsolateral prefrontal cortex, *CN* caudate nucleus, *PCC* posterior cingulate cortex

† Thal phasing of amyloid-β deposition phase (from 0, to 5) was available for a subset of participants as indicated by the fraction

### Genotyping

Whole blood lymphocytes or frozen brain tissue was used to extract DNA and previously defined quality control (QC) measures were employed [40]. *APOE* genotyping was performed by investigators blinded to cohort data at Polymorphic DNA Technologies. The *APOE* gene was sequenced defining differential isoforms of *APOE*- ε2, *APOE*- ε3, and *APOE*- ε4 by codons 112 and 158 on exon 4.

### Neuropsychological composites

Neuropsychological testing details are previously published [6, 8, 9]. There are 19 neuropsychological tests across 5 cognitive domains (episodic, semantic, and working memory, visuospatial ability/perceptual orientation, and perceptual speed) used in the calculation of a composite global cognition variable in ROS/MAP. This variable is meant to represent a participant’s overall cognitive functioning. Raw scores from each test were converted to z-scores using the mean and standard deviation. The final composite score is calculated by converting each test within each domain to a z-score and averaging all z-scores.

### Final summary clinical diagnosis

A clinical diagnosis was made at each participant visit based on the combination of cognitive tests scores, clinical judgement by a neuropsychologist, and diagnostic classification by a clinician (neurologist, geriatrician, or geriatric nurse practitioner) as previously described [6, 8, 9]. Clinical diagnosis of AD or other dementia followed criteria suggested by the joint working group of the National Institute of Neurological and Communicative Disorders and Stroke and the Alzheimer’s Disease and Related Disorders Association (NINCDS/ADRDA). Diagnosis of mild cognitive impairment (MCI) was rendered for persons who are judged to have cognitive impairment by the neuropsychologist but are classified as not meeting criteria for dementia by the clinician. The final summary clinical diagnosis was made at the time of death, blinded to post-mortem data, based upon review of select clinical data from all years by a neurologist.

### Neuropathological measures

#### Core AD pathology

All neuropathological marker quantifications have been previously described [6, 8, 9]. Briefly, quantification of neuritic plaques and neurofibrillary tangles was based on silver staining of five brain regions (midfrontal cortex, midtemporal cortex, inferior parietal cortex, entorhinal cortex, and hippocampus) averaged to obtain a summary score of the overall burden. In addition, immunohistochemistry was performed to calculate semi-quantitative scores for both amyloid-β and phospho-tau abundance in the cortex using antibodies specific to Aβ_1-42_ and abnormally phosphorylated tau, AT8 epitope, respectively, based off the average of eight regions (hippocampus, entorhinal cortex, midfrontal cortex, inferior temporal cortex, angular gyrus, calcarine cortex, anterior cingulate cortex, and superior frontal cortex). Braak staging of neurofibrillary tangle distribution and severity along with complimentary measures of neuritic plaque abundance by Consortium to Establish a Registry for Alzheimer’s disease (CERAD) scoring and spatial–temporal Aβ deposition capturing both neuritic and diffuse plaque progression by Thal phasing were also included as neuropathological outcomes and described elsewhere [7, 12, 39].

#### Cerebrovascular pathology

Rating of large vessel cerebral atherosclerosis was performed by visual inspection of the vertebral, basilar, posterior cerebral, middle cerebral, and anterior cerebral arteries of the circle of Willis, as well as their proximal branches and graded based on severity including the number of arteries involved and extent of artery involvement (0 = no pathology, to 4 = severe pathology). Additionally, given visual identification of occlusion, an artery was bisected to assess the degree of occlusion which would then be incorporated into the final score [1]. Arteriolosclerosis severity was classified by a semi-quantitative grading scale (0 = no pathology, to 3 = severe pathology) after characterization of histologic changes in the vascular lumen. These changes included but were not limited to the following in small vessels: intimal deterioration, smooth muscle degeneration, and fibrohyalinotic thickening of arterioles with consequent narrowing of the vascular lumen [13]. Macro infarcts were visualized on fixed slabs and dissected for confirmation [2, 46]. Microinfarcts were examined on 6 µm paraffin-embedded sections, stained with hematoxylin/eosin. Gross and micro-infarcts were categorized as present (1) or absent (0) based upon visual inspection in nine brain regions (midfrontal, middle temporal, entorhinal, hippocampal, inferior parietal and anterior cingulate cortices, anterior basal ganglia, midbrain, and thalamus) [2]. A semi-quantitative score for cerebral amyloid angiopathy (CAA) was measured by amyloid-β immunostaining in neocortical regions (midfrontal, midtemporal, angular, and calcarine cortices), and was scored on a scale from 0 to 4 (0 = no pathology, 4 = severe pathology). For each brain region, a meningeal and parenchymal vessel score was obtained, and the maximum of these was then used in each case. Final scores were averaged across regions [10].

### Autopsy measures of TREM2 mRNA expression

A standard post-mortem biological specimen protocol was utilized across centers where autopsies were performed and has been previously described [8]. RNA extraction from individual brain regions was performed using a Qiagen miRNeasy mini kit and a RNase free DNase Set for quantification on a Nanodrop. An Agilent Bioanalyzer assessed integrity and purity. A RIN score greater than five was used as inclusion criteria for next-generation RNA sequencing in bulk.

Sequencing was carried-out in multiple phases; phase one included the dorsolateral prefrontal cortex (dlPFC) while phase two added additional dlPFC samples as well as samples from the posterior cingulate cortex (PCC) and head of the caudate nucleus (CN). Phase three ran additional participant samples from the dlPFC. Detailed information on RNA processing and sequencing can be found on synapse (syn3388564). In summary, phase one utilized poly-A selection, strand-specific dUTP library preparation, and Illumina HiSeq with 101 bp paired-end reads achieving a coverage of 150 million reads of the first 12 reference samples. The deep sequencing of these 12 reference samples included 2 males and 2 females of non-impaired, mild cognitive impaired, and Alzheimer’s disease cases. The remaining samples underwent sequencing with a coverage of 50 million reads. Phase two, library preparation utilized KAPA Stranded RNA-Seq Kit with RiboErase (kapabiosystems) with which ribosomal depletion and fragmentation was performed. Sequencing of this phase was performed on an Illumina NovaSeq6000 using 2 × 100 bp cycles targeting 30 million reads per sample. Phase three, RNA was extracted with a Chemagic RNA tissue kit (Perkin Elmer, CMG-1212) using a Chemagic 360 instrument and ribosomal RNA was depleted using RiboGold (Illumina, 20,020,599). Sequencing of phase three was performed on an Illumina NovaSeq6000 with 40-50 M 2 × 150 bp paired-end reads.

Data processing and QC of RNA sequencing runs was performed by the Vanderbilt Memory and Alzheimer’s Center Computational Neurogenomics Team using an automated pipeline [49]. This included harmonizing the three dlPFC phases (*N* = 631, *N* = 278, and *N* = 104, 1–3, respectively) and independently processing the other brain regions (PCC; *N* = 571 and CN; *N* = 745). In brief, all samples with a RIN score below 4 and/or a post-mortem interval greater than 24 h were removed. Alignment was made to the hg38 reference genome using STAR. Read counts per million (CPM) values were quantile normalized to adjust for global variability between samples using the R package, *cqn* (version 1.30.0), controlling for GC-content and gene length. The R package, *limma*, was used to adjust for batch effects. In addition, principal component outliers outside five standard deviations as well as genes missing covariates of interest or those necessary for normalization were removed. Statistical outliers of gene expression outside four standard deviations from the mean were removed.

### Cellular fraction data

A deconvolution technique was previously employed to obtain cellular fraction data on a subset of ROS/MAP participants [38]. This method consisted of a subset of bulk RNA samples from the dlPFC also having single-nucleus data (*N* = 48 individuals and 80,660 single-nucleus transcriptomes) which was used to find the best predictors of each cellular component (i.e., excitatory neuron, microglia, oligodendrocyte, etc.) using all of the genes in the RNAseq data to build models with the most optimized set of genes. The isolation and extraction of nuclei from frozen tissue has been described previously [25]. Briefly, analysis of single-nucleus data (snRNA-seq) followed high-throughput droplet technology and massively parallel sequencing following the DroNc-seq protocol [25] with modification for the 10 × Genomics Chromium platform. Gene counts were obtained by alignment of reads to the hg38 reference genome (GRCh38.p5) using CellRanger software. Unspliced nuclear transcripts were accounted for by counting reads mapped to pre-mRNA. Each individual library was quantified for pre-mRNA and then aggregated to equalize read depth between libraries to generate a gene count matrix.

Quality control for cell inclusion has been previously described in detail [38]. The final dataset consisted of 17,926 genes in 75,060 nuclei. This snRNA-seq data were utilized in a regression-based approach for the generation of a reference expression profile and decomposition of bulk RNA sequencing data yielding cellular fraction estimates for each sample across eight cell types (microglia, astrocytes, inhibitory neurons, excitatory neurons, oligodendrocytes, oligodendrocyte progenitors, and endothelial or pericyte cells).

### Microglial density data

Microglial density measurements were previously obtained from brain samples using immunohistochemistry performed by an Automated Leica Bond immunostainer (Leica Microsystems Inc.) and anti-human HLA-DP, DQ, and DR antibodies (clone CR3/43; DakoCytomation; 1:100) [19]. A blinded investigator sampled 4% of an ROI with fixed magnification (400x) marking microglia counts and identifying their phenotypic activation stage. Stage 1 being least or not activated and having thin, ramified processes. Stage 2 being activated with a rounded cell body > 14um in size with thickened processes. Stage 3 being activated with criteria as in stage 2 but in addition having a macrophage appearance. Total counts for different stages were counted separately from adjacent blocks of tissue (0.5–1 cm apart) then summed, divided by the area of the sample, and multiplied by 10^6^ yielding a composite average density.

### Statistical analyses

Statistical analyses were performed in R v3.6.1 using R Studio IDE (https://www.rstudio.com/). To evaluate the data, a multiple linear regression model (for cross-sectional cognition and AD-related pathology) as well as a linear mixed-effects model (for longitudinal cognition) were used. Models were run separately by regional *TREM2* expression. For binomial and multinomial cerebrovascular outcome variables, generalized linear and proportional odds models were substituted, respectively. Linear regression models covaried for age at death, sex, post-mortem interval, education, and the interval from the last visit to death. In mixed-effects regression models, time was modeled as years from final visit with an additional covariate for the time between final visit and death. Both time and intercept were entered as fixed and random effects in the model. Immunohistochemistry and silver staining measurements of AD pathology were square root transformed to better approximate a normal distribution. Secondary analyses were conducted to account for possible variation in model predictions due to microglial cell-type fraction by including this estimate as a covariate as well as analyses assessing differences in results between AD cases and controls by stratifying the data.

All primary models were corrected for multiple comparisons using the Benjamini and Hochberg (1995) false discovery rate based on the total number of tests completed, accounting for all gene-tissue combinations across main effect models assessing neuropathological outcome measures (*N* = 30). Among the 945 participants with *TREM2* mRNA measurement from the dlPFC, 505 participants also had *TREM2* measurement from the PCC and 670 participants also had measurement from the CN. There were 430 participants with *TREM2* measurement from all three brain regions.

## Results

### Participant demographics

Participant characteristics are summarized in Table 1. The percentage of male compared to female participants was significantly less in all tissue cohorts. Participants were long-lived (mean age at death > 89 years), predominantly non-Hispanic white (≥ 98%) and female (> 65%) and were highly educated (mean > 16 years of education). The percentage of participants with greater severity and/or progression of neuropathology as measured by Braak staging, Thal phasing, or CERAD scoring was similar across brain regions.

### TREM2 mRNA expression across demographic characteristics

**Fig. 1:**
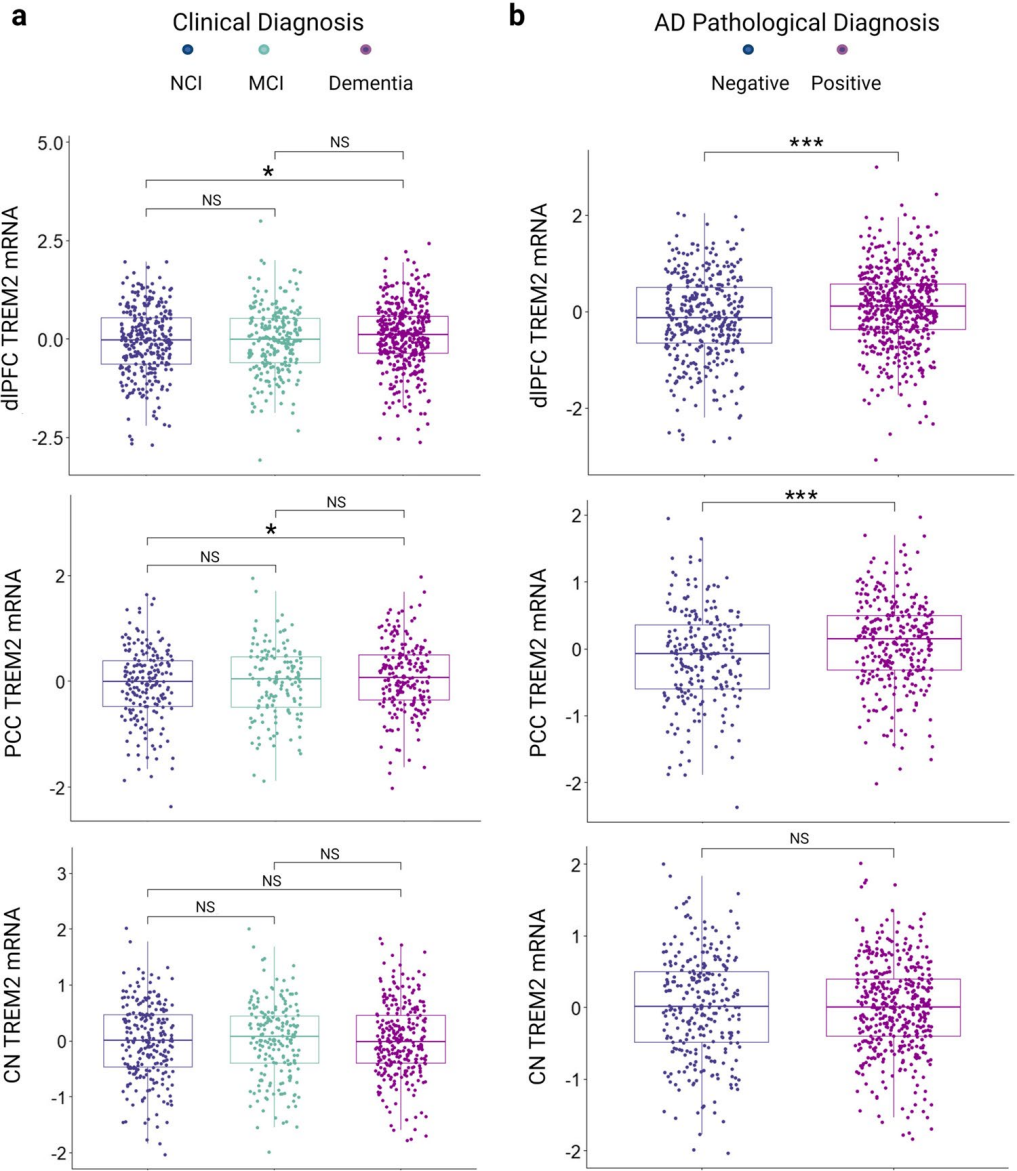
*TREM2 *expression across diagnosis. Cortical *TREM2* expression is higher in AD compared to control while caudate *TREM2* expression does not differ across diagnosis. **a** Final summary clinical diagnosis; no cognitive impairment (NCI), mild cognitive impairment (MCI) and Alzheimer’s disease dementia (AD). **b** Pathological diagnosis according to neuropathologic staging (CERAD and Braak) NIA-Reagan criteria. Positive; AD present (high or intermediate likelihood) and Negative; AD not present (low likelihood or no AD). Significance values are derived from the results of student’s *t*-tests. NS: *p* > 0.05. *p ≤ 0.05, **p ≤ 0.01, ***p ≤ 0.001

*TREM2* mRNA in the dlPFC and PCC was higher in individuals with a clinical diagnosis of AD compared to those with no cognitive impairment (NCI) (Fig. 1A; *p* = 0.030, and *p* = 0.043, respectively) but no different across *APOE*-ε4 carrier status (Supplementary Figure 1A-B; *p* = 0.095 and *p* = 0.071, respectively). Levels of *TREM2* mRNA in the CN neither differed across clinical diagnosis nor *APOE*-ε4 carrier status (Fig. 1A and Supplementary Figure 1C). When restricting to autopsy confirmed AD diagnosis, dlPFC and PCC *TREM2* levels were higher in individuals with neuropathologically confirmed clinical AD (high or intermediate likelihood of AD) as compared to NCI individuals without or with low likelihood of AD as classified by NIA-Reagan criteria (Fig. 1B; *p* = 8.6e-05, and *p* = 3.9e-4, respectively). In contrast, caudate *TREM2* levels did not differ across clinical or neuropathologically confirmed AD (Fig. 1B; *p* = 0.780). As expected, *TREM2* mRNA was found to be significantly correlated with the microglial cell-type fraction compared to other fractions (Supplementary Figure 2; representative data from dlPFC, *r* = 0.7).

### TREM2 mRNA associations with amyloid

**Fig. 2:**
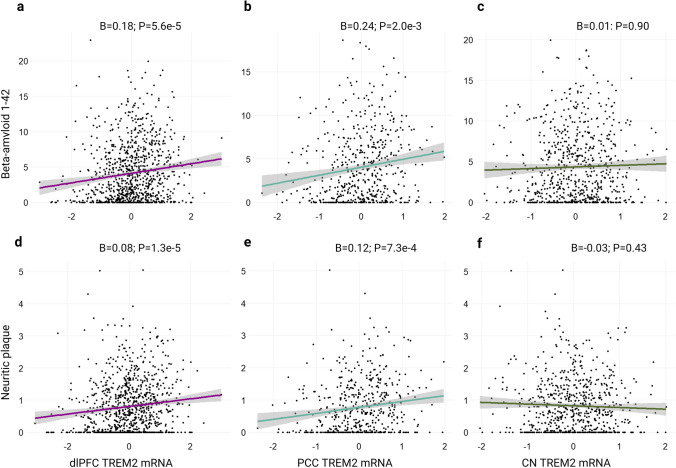
*TREM2* associations with amyloid. Cortical but not caudate *TREM2* mRNA positively associates with amyloid neuropathology. **a**–**c** Regional *TREM2* mRNA levels by Aβ1-42 burden as measured by immunohistochemistry. **d**–**f** Regional *TREM2* mRNA levels by neuritic plaque burden as measured by silver stain. Unadjusted scatter plots and statistical results from linear regression models adjusting for age at death, sex, education, and post-mortem interval

As expected, *TREM2* mRNA levels were associated with higher amyloid burden in both cortical regions and across both amyloid measures (Table 2 and Fig. 2). *TREM2* levels in the CN were not associated with either amyloid outcome measure (Table 2 and Fig. 2). Results were similar when models were adjusted for microglial fraction (Supplementary Table 1). Positive cortical associations were largely driven by AD cases (Supplementary Figure 3A, B, D and E), while there were notable negative associations between caudate *TREM2* and amyloid in AD cases only (Supplementary Figure 3C, F). Main effect results per regional measurement of amyloid yielded similar outcomes and are provided in Supplementary Table 2. PCC and dlPFC but not CN *TREM2* expression was higher in participants with higher amyloid burden as determined by CERAD scoring (Supplementary Figure 4). In contrast, when assessed across Thal phases considering spatial–temporal distribution, there are significant increases in PCC *TREM2* only (Supplementary Figure 5).

**Table 2:** Main effects of *TREM2* on amyloid

Predictor (Tissue)	Outcome	*β*	SE	*P* value	P.fdr
dlPFC	Aβ_1-42_	0.179	0.044	**5.62e-05**	**1.69e-03**
dlPFC	Neuritic plaque	0.080	0.021	**1.28e-05**	**1.92e-03**
PCC	Aβ_1-42_	0.236	0.073	**0.002**	**0.016**
PCC	Neuritic plaque	0.118	0.035	**7.27e-04**	**7.27e-03**
CN	Aβ_1-42_	0.012	0.066	0.852	0.896
CN	Neuritic plaque	− 0.030	0.030	0.330	0.459

Boldface signifies *p* < 0.05

*dlPFC* Dorsolateral prefrontal cortex, *CN* caudate nucleus, *PCC* posterior cingulate cortex

### TREM2 mRNA associations with Tau

**Fig. 3:**
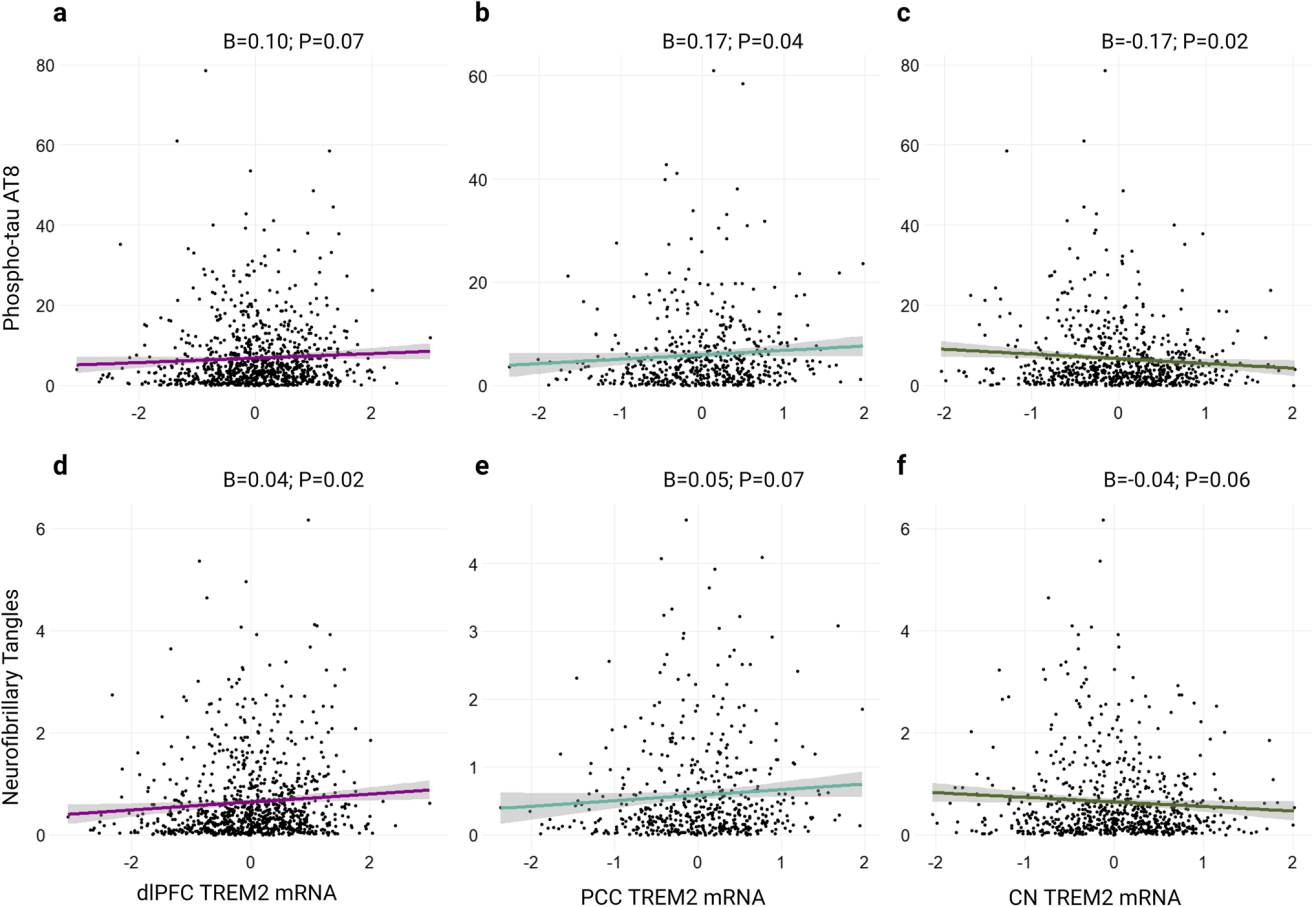
*TREM2* associations with tau. *TREM2* mRNA associations with tau are weak in primary analyses of unstratified data. **a**–**c** Regional *TREM2* mRNA levels by phosphorylated tau (AT8 epitope, Ser202/Thr305) burden as measured by immunohistochemistry. **d**–**f** Regional *TREM2* mRNA levels by neurofibrillary burden as measured by silver stain. Unadjusted scatter plots and statistical results from linear regression models adjusting for age at death, sex, education, and post-mortem interval

Primary association results across the entire cohort for *TREM2* mRNA expression and tau neuropathology were weak. *TREM2* mRNA was negatively associated with tau burden in the CN, albeit nominally (Fig. 3C; *β* = − 0.17; *p* = 0.02), and positively associated with the two cortical regions (Fig. 3A, B, D and E). However, Table 3 shows that these results do not survive correction for multiple comparisons.

**Table 3:** Main effects of *TREM2* on Tau

Predictor (Tissue)	Outcome	B	SE	*P* value	P.fdr
dlPFC	p-Tau, AT8	0.095	0.052	0.069	0.147
dlPFC	neurofibrillary tangles	0.039	0.016	**0.016**	0.061
PCC	p-Tau, AT8	0.168	0.083	**0.043**	0.118
PCC	neurofibrillary tangles	0.047	0.026	0.074	0.147
CN	p-Tau, AT8	− 0.173	0.075	**0.020**	0.061
CN	neurofibrillary tangles	− 0.044	0.024	0.062	0.142
Models Adjusting for Aβ1-42 level					
dlPFC	neurofibrillary tangles	0.009	0.014	0.546	0.630
PCC	p-Tau, AT8	0.053	0.075	0.478	0.594
CN	p-Tau, AT8	− 0.180	0.067	**0.008**	**0.045**

Boldface signifies *p* < 0.05

*dlPFC* Dorsolateral prefrontal cortex, *CN* caudate nucleus, *PCC* posterior cingulate cortex

In post-hoc models adjusting for Aβ_1-42_ levels, cortical *TREM2* associations with tau were attenuated, while the caudate *TREM2* association became significant (Table 3). When plotting the effect in the CN, it appears that *TREM2* associations with tau burden may depend on disease state whereby the association is present in amyloid positive but not amyloid negative individuals (Supplementary Figure 6) and AD cases but not controls (Supplementary Figure 7). Tau results were similar in sensitivity analyses adjusting for microglial fraction (Supplementary Table 3). Main effect results per regional measurement of phosphorylated tau are provided in Supplementary Table 2. Subtle increases in cortical *TREM2* levels were observed across Braak staging supporting the linear regression results with tau (Supplementary Figure 8A, B). Interestingly, decreases in CN *TREM2* mRNA are shown in later Braak stages which may help explain the negative model estimates in this region (Supplementary Figure 8C).

### TREM2 mRNA associations with cerebrovascular pathology

Despite the initial hypothesis that upregulation of *TREM2* mRNA at autopsy may reflect concomitant cerebrovascular pathology, this was largely unsupported by results using cross-sectional data from ROS/MAP. Supplemental Table 4 summarizes the main effects analyses. Cell-type sensitivity analyses using dlPFC data yielded insignificant results and are reported in Supplementary Table 5.

### TREM2 associations with microglial density and activation

**Fig. 4:**
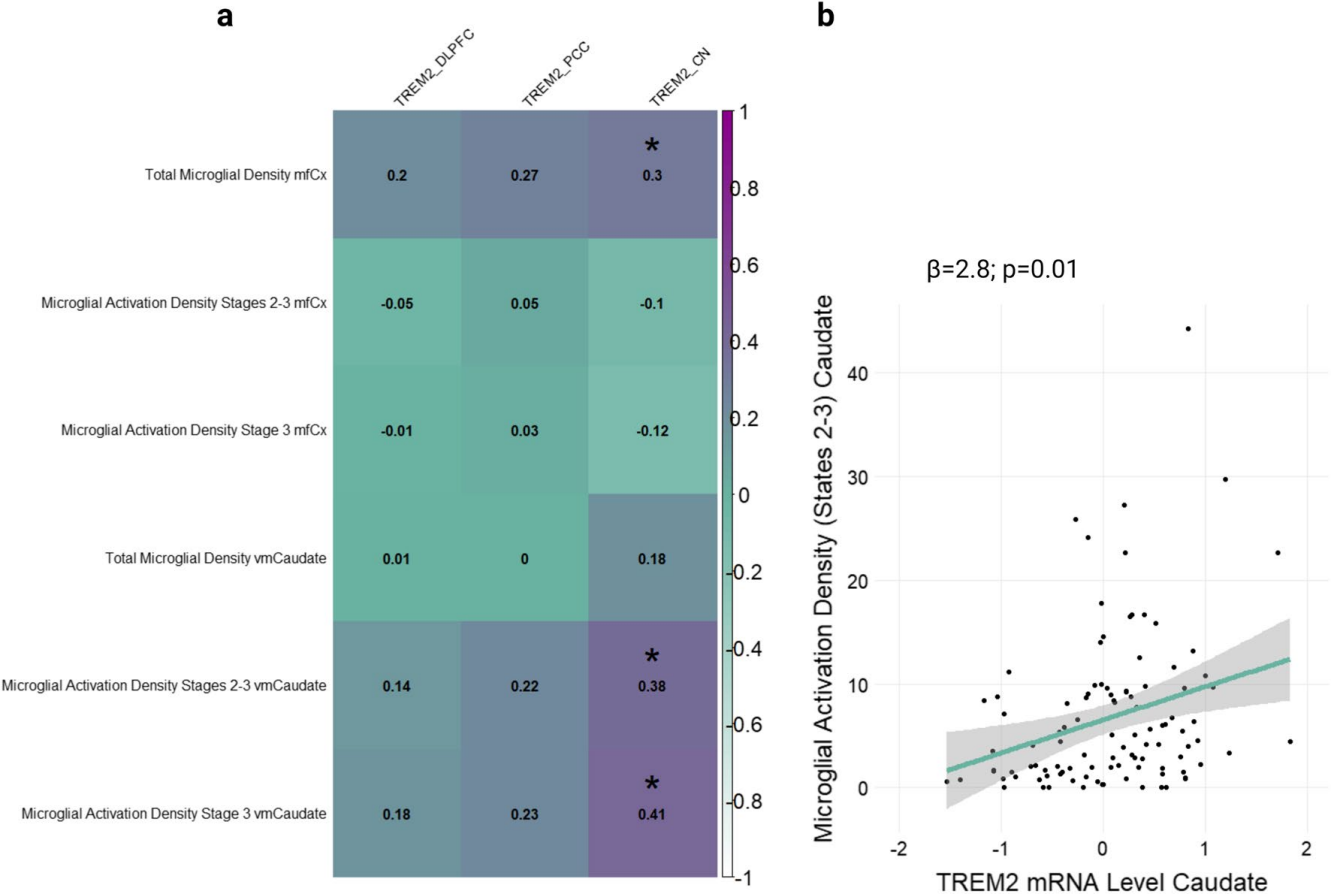
*TREM2* correlations with microglial density components. **a** Caudate but not cortical *TREM2* mRNA is significantly correlated with several components of microglial density including the activated component cis-regionally. Midfrontal cortex (mfCx); ventral medial caudate (vmCaudate). A Pearson’s correlation coefficient (r) is displayed for each comparison. An asterisk denotes significance set to an a priori threshold of *p* < 0.05. **b** Caudate *TREM2* mRNA levels by the proportion of activated microglial density (PAM) stages 2–3. Unadjusted scatter plots and statistical results from linear regression models adjusting for age at death, sex, education, and post-mortem interval

Next, it was investigated whether the associations between *TREM2* and AD neuropathology described above were accompanied by *TREM2* associations with microglial density and/or activation in the same regions. A subset of participants (*N* = 156 cortical and *N* = 104 caudate) had transcript and microglial activation data available for analysis (see Supplementary Table 6 for participant characteristics). Interestingly, *TREM2* measurements from cortical regions were not correlated with microglial outcome measures as we anticipated. However, *TREM2* levels were significantly correlated with the activated component of microglial density cis-regionally in the caudate (Fig. 4A). This association survived adjustment for covariates in a multiple linear regression model (stages 2–3 caudate microglia ~ age at death + sex + education + post-mortem interval, + caudate TREM2; *β* = 2.84, se = 1.10, *p* = 0.011; Fig. 4B), but did not survive correction for multiple comparisons. Diagnosis-stratified results show that this association is significant in AD cases but not controls, suggesting caudate microglial dynamics may change in AD (Supplementary Figure 9). A scatter plot of CN *TREM2* and PAM stratified by diagnosis is provided in Supplementary Figure 10. Next, it was investigated whether levels or severity of concomitant neuropathology interacted with *TREM2* expression on this activated microglial state. There was a lack of evidence that select AD neuropathology, including morphological substrates of small vessel disease, modified the association between *TREM2* and microglial activation density in the caudate suggesting this signal may represent a collection of diverse biological processes beyond the scope of individual AD neuropathologies examined herein (Supplementary Table 7).

**Fig. 5:**
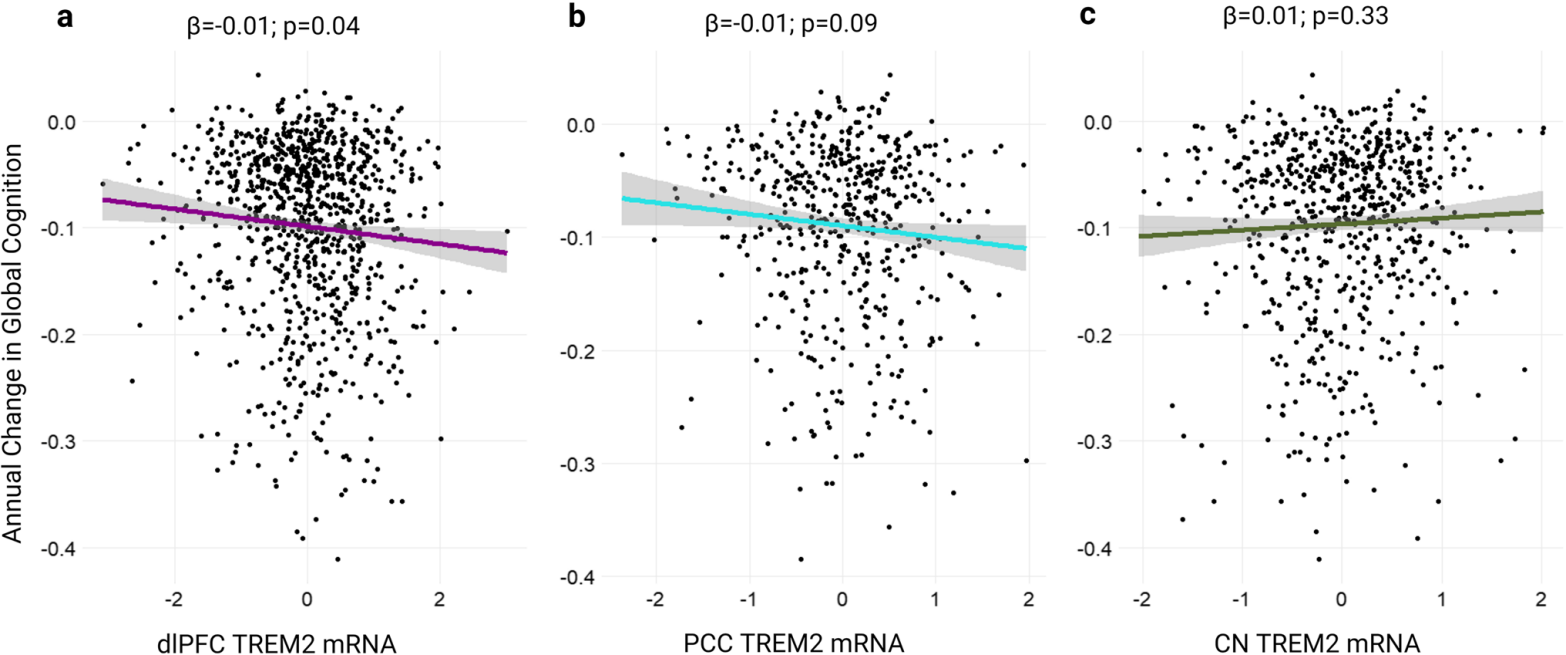
*TREM2* associations with global cognition. *TREM2* cortical but not caudate mRNA at autopsy is a predictor of retrospective global cognitive decline in primary unstratified analyses. Unadjusted scatter plots and statistical results from linear regression models adjusting for age at death, sex, education, phosphorylated tau level, and post-mortem interval. **a** Dorsolateral prefrontal cortex (dlPFC) *TREM2* mRNA levels by annual change in global cognitive performance. **b** Posterior cingulate cortex (PCC) *TREM2* mRNA levels and **c** head of caudate nucleus (CN) *TREM2* mRNA levels

Next, we explored potential *TREM2* mRNA expression associations with composite global cognition scores. Cross-sectional results revealed *TREM2* mRNA levels were not significantly associated with cognition prior to death in any brain region (*p* > 0.15). However, *TREM2* mRNA levels related to faster decline in longitudinal analysis from the dlPFC (Fig. 5A; *p* = 0.04). *TREM2* mRNA levels in the PCC and CN were not significantly associated with longitudinal global cognition (Fig. 5B, C; *p* = 0.09 and *p* = 0.97, respectively). Interestingly, diagnosis-stratified graphs revealed positive relationship between *TREM2* and cognition in AD cases only when *TREM2* was measured from the CN (Supplementary Figure 11C).

Finally, we tested whether amyloid status interacted with *TREM2* levels on longitudinal cognition scores. *TREM2* levels did not significantly interact with amyloid positivity on cognitive trajectory (*p* > 0.08), plots stratified by amyloid status are presented in Supplementary Figure 12.

## Discussion

This work evaluated the effects of *TREM2* gene expression on AD neuropathology, cognition, and concomitant pathways of injury in the human brain. We found striking evidence that *TREM2* transcript associations are region-specific, with high cortical *TREM2* relating to both amyloid pathology and clinical AD, while high caudate *TREM2* was associated with microglial activation, less AD neuropathology, and a slower rate of global cognitive decline in AD cases. These findings highlight the regional complexity of *TREM2* and suggest that changes in cortical TREM2 signaling may be particularly relevant to amyloid deposition while changes in caudate TREM2 signaling may be relevant at the onset of AD diagnosis.

Amongst neuropathology outcome measures, perhaps the most robust was the association between *TREM2* and Aβ burden. This is consistent with the fact that functional responses of microglia to plaque have been shown to reflect increases in transcription of *TREM2* which, in turn, is vital to microglial barrier formation and subsequent compaction of the plaque residue. These associations were consistent in cortical brain tissues and supported by previous work showing post-mortem immunoreactivity of cortical *TREM2* in microglia, particularly those surrounding plaque [36]. Furthermore, in APP23 transgenic mice, microglia surrounding Aβ increased expression of *TREM2* corresponding with the progression of amyloid pathology [21]. The CN is also a region shown to be vulnerable to amyloid pathology, albeit a subcortical region thought to be affected later-on in the spatial–temporal pattern of plaque progression in AD as compared to neocortical regions [23]. Yet, *TREM2* mRNA levels from the CN did not relate to Aβ burden (Fig. 5). This is perhaps explained by the methods of amyloid quantification which incorporated an average amyloid burden score from mostly cortical subregions without any calculations from the basal ganglia. Therefore, subcortical *TREM2* expression did not predict Aβ pathology transregionally. This lack of association may be explained by the lack of neuritic plaques within the caudate relative to the cortex [4, 11, 54]. That said, *TREM2* levels in the caudate were relatively similar across Thal stages (which include basal ganglia; Supplementary Figure 5C) and across CERAD scores (Supplementary Figure 4C).

Cortical *TREM2* expression also related to cognitive decline, although this association was weak and limited to the dlPFC in primary analyses. We expand upon this result by showing that the association with cognition is likely related to neuropathological tissue status, as differences between AD cases and controls were most pronounced when using a neuropathologically confirmed diagnosis (Fig. 2B). If higher levels in *TREM2* mRNA reflect a microglial-mediated response to neuropathology, then the association between *TREM2* and cognition is likely driven by individuals with a higher burden of neuropathology. While our amyloid status-stratified plots further support this interpretation (Supplementary Figure 12), it should be noted that we did not observe a statistically significant interaction between amyloid burden and *TREM2* abundance on cognitive decline. Ultimately further work is needed, perhaps focusing on cell-specific expression, to better understand how and when *TREM2* changes with disease.

To better understand how other microglial inflammatory markers relate to amyloid, we employed a post-hoc analysis of a small panel of transcripts (*CD44*, *CD45*, *CD11B*, *SPP1*, and *LGALS3*) and observed that their expression levels, like those of *TREM2*, differ regionally; more pronounced positive associations were found in the dlPFC (with the exception of *LGALS3*), while none of these markers were significantly associated with amyloid when measured from the CN (Supplementary Table 8). *TREM2* and other microglial transcripts were positively correlated amongst each other regardless of region (Supplementary Figure 13). These results highlight the need to further investigate differences in microglial activation and subpopulation heterogeneity using multiple markers between cortical and subcortical brain regions and their potential differing roles in neurodegeneration in the presence of amyloid.

In contrast to amyloid, there was weaker evidence of cortical *TREM2* expression association with tau burden in primary unstratified analyses. In fact, there is a flip in the direction of association of *TREM2* with tau when *TREM2* is measured from caudate tissue (Fig. 3C). This result mirrors the same flip in direction between caudate *TREM2* and amyloid (Supplementary Figure 3C, F) as well as caudate *TREM2* and cognition (Supplementary Figure 11C), when the associations are limited to AD cases only, suggesting the negative relationship between caudate *TREM2* and neuropathology may be relevant in disease. However, these stratified results need replication in an independent cohort. Previous work has found a similar positive association between *TREM2* expression in human temporal cortex and phosphorylated tau [36]. This may be explained by several factors. Unlike the direct functional relationship between TREM2 binding Aβ ligand [62], there is currently a lack of evidence of a direct functional relationship between TREM2 and phosphorylated tau or other intraneuronal pathological process leading to dystrophic neurite formation. Rather, it is thought to be the case that TREM2 regulation of microglial activity and subsequent immune dysfunction are external triggers of neuronal tau pathology via microgliosis and excessive neuroinflammation [35]. Furthermore, the dynamic between *TREM2* transcriptional upregulation and Aβ deposition is thought to be deleterious to microglia and the immune landscape, whereas the development of tau pathology downstream has been shown to be dependent to some extent on a priori amyloid accumulation [41]. Therefore, transcriptional upregulation of *TREM2* mRNA expression may be closer coupled to levels of amyloid rather than tau pathology in earlier affected cortical regions; this is despite concurrent elevations in its soluble protein fragment and phosphorylated tau in CSF described in MCI and early AD [27, 51]. In contrast were the notable associations of high caudate *TREM2* and low tau pathology in AD-stratified data (Supplementary Figure 7C , F) lending the possibility that subpopulations of microglia residing in this subcortical region may have a beneficial role staving off neurodegeneration as disease progresses. Our results highlight the possibility that *TREM2* associations with tau may vary by brain region and disease state, though more confirmatory work is needed.

We provide modest evidence that cortical *TREM2* expression relates to amyloid while caudate *TREM2* expression, instead, relates to microglial reactivity. This is interesting given that it is not explicitly clear whether there are disparate functions of TREM2 in microglia derived from these distinct brain regions. Evidence from transcriptomic- and other omic-profiling has revealed distinctive spatial and molecular patterns of microglia across the brain [32, 37, 52]. And recent work highlights regional pathology-associated differences between cortical and striatal glial transcript expression [60]. Although our post hoc interaction models suggest a lack evidence for AD neuropathology interactions on this association with PAM in the caudate, diagnosis-stratified results suggest this is indeed an AD-relevant phenomenon—it may be that our analyses are picking up on a compensatory response of the TREM2 pathway in the caudate that is disease-stage specific (see *TREM2* across Braak stages Supplementary Figure 8C, phospho-tau levels by amyloid status Supplementary Figure 6, and annual changes in global cognition stratified by diagnosis Supplementary Figure 11C). Due to the finding that increased *TREM2* in caudate relates to the microglial state cis-regionally, we are left to speculate as to why this association does not extend to the cortex (Fig. 4). There are several possibilities for this including limitations to using bulk RNA sequencing measurement of *TREM2* transcript abundance which may obscure cell-specific or isoform-specific resolutions. The lack of association signal in the cortex between microglial activation and *TREM2* could otherwise be due to a window of late-life measurement when *TREM2* transcription may not reflect a microglial activation response. For example, Aβ plaque deposits more abundantly in the cortex of AD patients [47] and previous studies have shown microglial metabolism may become inefficient or fail given a high amyloid burden [3] which paves the possibility that the lack of association in this region may be due to loss of function. This is particularly intriguing given the recent links between microglial metabolic fitness, glucose metabolism, and TREM2 function [33, 55]. The lack of robust findings using cerebrovascular outcome measures suggests that upregulation of *TREM2* in late life may be a specific response to the abnormal accumulation of classical AD neuropathology.

There are several strengths and limitations to the present analyses. First, ROS/MAP is a well-characterized longitudinal cohort with deep clinical and neuropathological measures. Second, the availability of longitudinal cognitive data and *TREM2* measurement in multiple brain regions bolstered characterizations. However, analyses herein are limited to a late-life window of neuropathology at autopsy, therefore, *TREM2* expression changes in brain throughout the course of normal aging and disease in humans remain unknown, begging the advent of a TREM2-specific PET radioligand assay. Moreover, measurement of RNA transcript does not necessarily translate to protein expression or signaling competent membranous TREM2. Potential discordance between mRNA and protein expression may be especially true for dystrophic cell types and during severe disease, therefore, results may not reflect true microglial activity [18, 53]. A limitation of our gene expression associations with neuropathology is that neuropathology was not quantified in identical brain regions. While AD neuropathology largely follows a well-characterized spatial pattern across disease stages, it is possible that we are missing some region-specific effects due to the lack of amyloid and tau measurement in the caudate and posterior cingulate region. Finally, the lack of racial diversity precludes generalization of results to more diverse populations. Future work, looking at whether caudate *TREM2* associations with Aβ and or tau are present if measured cis-regionally are needed to clarify disparate regional results herein.

Taken together, data support previous preclinical demonstrations of a strong functional relationship between TREM2 protein expression and amyloid which is evident in the present cohort at autopsy. Secondary analysis using AD diagnosis-stratified data showed *TREM2* mRNA expression associations with tau neuropathology in AD cases but not controls, suggesting transcriptional upregulation of *TREM2* is a disease-relevant response to neurodegeneration. Finally, we draw attention to a previously unrecognized difference between cortical and caudate *TREM2* expression associations; the former with increased classical AD neuropathology and cognitive decline, and the latter with decreased classical AD neuropathology, a less rapid cognitive decline, and microglial activation. We posit that these regional differences may be due, at least in part, to relatively spared microglial function in the caudate compared to earlier-affected cortical regions. Results suggest that cortical and caudate TREM2 signaling may have disparate roles in AD progression.

## Supplementary Information

Below is the link to the electronic supplementary material.

Supplementary file1 (DOCX 2517 KB)

## Data Availability

ROS/MAP data can be requested by qualified investigators (www.radc.rush.edu).

## Abbreviations

TREM2: Triggering receptor expressed on myeloid cells-2
AD: Alzheimer’s disease
MCI: Mild cognitive impairment
ROS/MAP: Religious orders study and rush memory and aging project
Aβ: Amyloid-beta
*APOE*-ε4: Apolipoprotein E epsilon 4
dlPFC: Dorsolateral prefrontal cortex
PCC: Posterior cingulate cortex
CN: Head of caudate nucleus
PAM: Proportion of activated microglia

## References

[1] Arvanitakis Z, Capuano AW, Leurgans SE, Buchman AS, Bennett DA, Schneider JA (2017). The relationship of cerebral vessel pathology to brain microinfarcts. Brain Pathol.

[2] Arvanitakis Z, Leurgans SE, Barnes LL, Bennett DA, Schneider JA (2011). Microinfarct pathology, dementia, and cognitive systems. Stroke.

[3] Baik SH, Kang S, Lee W, Choi H, Chung S, Kim JI, Mook-Jung I (2019). a breakdown in metabolic reprogramming causes microglia dysfunction in Alzheimer's disease. Cell Metab.

[4] Beach TG, Sue LI, Walker DG, Sabbagh MN, Serrano G, Dugger BN, Mariner M, Yantos K, Henry-Watson J, Chiarolanza G (2012). Striatal amyloid plaque density predicts Braak neurofibrillary stage and clinicopathological Alzheimer's disease: implications for amyloid imaging. J Alzheimers Dis.

[5] Bekris LM, Khrestian M, Dyne E, Shao Y, Pillai JA, Rao SM, Bemiller SM, Lamb B, Fernandez HH, Leverenz JB (2018). Soluble TREM2 and biomarkers of central and peripheral inflammation in neurodegenerative disease. J Neuroimmunol.

[6] Bennett DA, Buchman AS, Boyle PA, Barnes LL, Wilson RS, Schneider JA (2018). Religious orders study and rush memory and aging project. J Alzheimers Dis.

[7] Bennett DA, Schneider JA, Arvanitakis Z, Kelly JF, Aggarwal NT, Shah RC, Wilson RS (2006). Neuropathology of older persons without cognitive impairment from two community-based studies. Neurology.

[8] Bennett DA, Schneider JA, Buchman AS, Barnes LL, Boyle PA, Wilson RS (2012). Overview and findings from the rush memory and aging project. Curr Alzheimer Res.

[9] Bennett DA, Wilson RS, Arvanitakis Z, Boyle PA, de Toledo-Morrell L, Schneider JA (2013). Selected findings from the religious orders study and rush memory and aging project. J Alzheimers Dis.

[10] Boyle PA, Yu L, Nag S, Leurgans S, Wilson RS, Bennett DA, Schneider JA (2015). Cerebral amyloid angiopathy and cognitive outcomes in community-based older persons. Neurology.

[11] Braak H, Braak E (1990). Alzheimer's disease: striatal amyloid deposits and neurofibrillary changes. J Neuropathol Exp Neurol.

[12] Braak H, Braak E (1991). Neuropathological stageing of Alzheimer-related changes. Acta Neuropathol.

[13] Buchman AS, Leurgans SE, Nag S, Bennett DA, Schneider JA (2011). Cerebrovascular disease pathology and parkinsonian signs in old age. Stroke.

[14] Cady J, Koval ED, Benitez BA, Zaidman C, Jockel-Balsarotti J, Allred P, Baloh RH, Ravits J, Simpson E, Appel SH (2014). TREM2 variant p. R47H as a risk factor for sporadic amyotrophic lateral sclerosis. JAMA Neurol.

[15] Carbajosa G, Malki K, Lawless N, Wang H, Ryder JW, Wozniak E, Wood K, Mein CA, Dobson RJB, Collier DA (2018). Loss of Trem2 in microglia leads to widespread disruption of cell coexpression networks in mouse brain. Neurobiol Aging.

[16] Celarain N, Sanchez-Ruiz de Gordoa J, Zelaya MV, Roldan M, Larumbe R, Pulido L, Echavarri C, Mendioroz M (2016). TREM2 upregulation correlates with 5-hydroxymethycytosine enrichment in Alzheimer's disease hippocampus. Clin Epigenetics.

[17] Davies DS, Ma J, Jegathees T, Goldsbury C (2017). Microglia show altered morphology and reduced arborization in human brain during aging and Alzheimer's disease. Brain Pathol.

[18] Ding Q, Markesbery WR, Chen Q, Li F, Keller JN (2005). Ribosome dysfunction is an early event in Alzheimer's disease. J Neurosci.

[19] Felsky D, Patrick E, Schneider JA, Mostafavi S, Gaiteri C, Patsopoulos N, Bennett DA, De Jager PL (2018). Polygenic analysis of inflammatory disease variants and effects on microglia in the aging brain. Mol Neurodegener.

[20] Felsky D, Roostaei T, Nho K, Risacher SL, Bradshaw EM, Petyuk V, Schneider JA, Saykin A, Bennett DA, De Jager PL (2019). Neuropathological correlates and genetic architecture of microglial activation in elderly human brain. Nat Commun.

[21] Frank S, Burbach GJ, Bonin M, Walter M, Streit W, Bechmann I, Deller T (2008). TREM2 is upregulated in amyloid plaque-associated microglia in aged APP23 transgenic mice. Glia.

[22] Gorenjak V, Aldasoro Arguinano AA, Dade S, Stathopoulou MG, Vance DR, Masson C, Visvikis-Siest S (2018). The polymorphism rs6918289 located in the downstream region of the TREM2 gene is associated with TNF-alpha levels and IMT-F. Sci Rep.

[23] Grothe MJ, Barthel H, Sepulcre J, Dyrba M, Sabri O, Teipel SJ, Alzheimer's Disease Neuroimaging I (2017). In vivo staging of regional amyloid deposition. Neurology.

[24] Guerreiro R, Wojtas A, Bras J, Carrasquillo M, Rogaeva E, Majounie E, Cruchaga C, Sassi C, Kauwe JS, Younkin S (2013). TREM2 variants in Alzheimer's disease. N Engl J Med.

[25] Habib N, Avraham-Davidi I, Basu A, Burks T, Shekhar K, Hofree M, Choudhury SR, Aguet F, Gelfand E, Ardlie K (2017). Massively parallel single-nucleus RNA-seq with DroNc-seq. Nat Methods.

[26] Hakola HP (1972). Neuropsychiatric and genetic aspects of a new hereditary disease characterized by progressive dementia and lipomembranous polycystic osteodysplasia. Acta Psychiatr Scand Suppl.

[27] Heslegrave A, Heywood W, Paterson R, Magdalinou N, Svensson J, Johansson P, Ohrfelt A, Blennow K, Hardy J, Schott J (2016). Increased cerebrospinal fluid soluble TREM2 concentration in Alzheimer's disease. Mol Neurodegener.

[28] Jay TR, von Saucken VE, Landreth GE (2017). TREM2 in neurodegenerative diseases. Mol Neurodegener.

[29] Jiang T, Tan L, Zhu XC, Zhou JS, Cao L, Tan MS, Wang HF, Chen Q, Zhang YD, Yu JT (2015). Silencing of TREM2 exacerbates tau pathology, neurodegenerative changes, and spatial learning deficits in P301S tau transgenic mice. Neurobiol Aging.

[30] Jiang T, Zhang YD, Chen Q, Gao Q, Zhu XC, Zhou JS, Shi JQ, Lu H, Tan L, Yu JT (2016). TREM2 modifies microglial phenotype and provides neuroprotection in P301S tau transgenic mice. Neuropharmacology.

[31] Jonsson T, Stefansson H, Steinberg S, Jonsdottir I, Jonsson PV, Snaedal J, Bjornsson S, Huttenlocher J, Levey AI, Lah JJ (2013). Variant of TREM2 associated with the risk of Alzheimer's disease. N Engl J Med.

[32] Keren-Shaul H, Spinrad A, Weiner A, Matcovitch-Natan O, Dvir-Szternfeld R, Ulland TK, David E, Baruch K, Lara-Astaiso D, Toth B (2017). A unique microglia type associated with restricting development of Alzheimer's disease. Cell.

[33] Kleinberger G, Brendel M, Mracsko E, Wefers B, Groeneweg L, Xiang X, Focke C, Deussing M, Suarez-Calvet M, Mazaheri F (2017). The FTD-like syndrome causing TREM2 T66M mutation impairs microglia function, brain perfusion, and glucose metabolism. EMBO J.

[34] Kleinberger G, Yamanishi Y, Suarez-Calvet M, Czirr E, Lohmann E, Cuyvers E, Struyfs H, Pettkus N, Wenninger-Weinzierl A, Mazaheri F (2014). TREM2 mutations implicated in neurodegeneration impair cell surface transport and phagocytosis. Sci Transl Med.

[35] Leyns CEG, Ulrich JD, Finn MB, Stewart FR, Koscal LJ, Remolina Serrano J, Robinson GO, Anderson E, Colonna M, Holtzman DM (2017). TREM2 deficiency attenuates neuroinflammation and protects against neurodegeneration in a mouse model of tauopathy. Proc Natl Acad Sci USA.

[36] Lue LF, Schmitz CT, Serrano G, Sue LI, Beach TG, Walker DG (2015). TREM2 protein expression changes correlate with Alzheimer's disease neurodegenerative pathologies in post-mortem temporal cortices. Brain Pathol.

[37] Masuda T, Sankowski R, Staszewski O, Bottcher C, Amann L, Sagar SC, Nessler S, Kunz P, van Loo G (2019). Spatial and temporal heterogeneity of mouse and human microglia at single-cell resolution. Nature.

[38] Mathys H, Davila-Velderrain J, Peng Z, Gao F, Mohammadi S, Young JZ, Menon M, He L, Abdurrob F, Jiang X (2019). Single-cell transcriptomic analysis of Alzheimer's disease. Nature.

[39] Mirra SS, Heyman A, McKeel D, Sumi SM, Crain BJ, Brownlee LM, Vogel FS, Hughes JP, van Belle G, Berg L (1991). The consortium to establish a registry for Alzheimer's Disease (CERAD). part II. Standardization of the neuropathologic assessment of Alzheimer's disease. Neurology.

[40] Oveisgharan S, Buchman AS, Yu L, Farfel J, Hachinski V, Gaiteri C, De Jager PL, Schneider JA, Bennett DA (2018). APOE epsilon2epsilon4 genotype, incident AD and MCI, cognitive decline, and AD pathology in older adults. Neurology.

[41] Pascoal TA, Benedet AL, Ashton NJ, Kang MS, Therriault J, Chamoun M, Savard M, Lussier FZ, Tissot C, Karikari TK (2021). Microglial activation and tau propagate jointly across Braak stages. Nat Med.

[42] Perez SE, Nadeem M, He B, Miguel JC, Malek-Ahmadi MH, Chen K, Mufson EJ (2017). Neocortical and hippocampal TREM2 protein levels during the progression of Alzheimer's disease. Neurobiol Aging.

[43] Poggesi A, Pasi M, Pescini F, Pantoni L, Inzitari D (2016). Circulating biologic markers of endothelial dysfunction in cerebral small vessel disease: a review. J Cereb Blood Flow Metab.

[44] Rayaprolu S, Mullen B, Baker M, Lynch T, Finger E, Seeley WW, Hatanpaa KJ, Lomen-Hoerth C, Kertesz A, Bigio EH (2013). TREM2 in neurodegeneration: evidence for association of the p.R47H variant with frontotemporal dementia and Parkinson's disease. Mol Neurodegener.

[45] Rosenberg GA, Wallin A, Wardlaw JM, Markus HS, Montaner J, Wolfson L, Iadecola C, Zlokovic BV, Joutel A, Dichgans M (2016). Consensus statement for diagnosis of subcortical small vessel disease. J Cereb Blood Flow Metab.

[46] Schneider JA, Bienias JL, Wilson RS, Berry-Kravis E, Evans DA, Bennett DA (2005). The apolipoprotein E epsilon4 allele increases the odds of chronic cerebral infarction [corrected] detected at autopsy in older persons. Stroke.

[47] Serrano-Pozo A, Frosch MP, Masliah E, Hyman BT (2011). Neuropathological alterations in Alzheimer disease. Cold Spring Harb Perspect Med.

[48] Serrano-Pozo A, Gomez-Isla T, Growdon JH, Frosch MP, Hyman BT (2013). A phenotypic change but not proliferation underlies glial responses in Alzheimer disease. Am J Pathol.

[49] Seto M, Weiner RL, Dumitrescu L, Mahoney ER, Hansen SL, Janve V, Khan OA, Liu D, Wang Y, Menon V (2022). RNASE6 is a novel modifier of APOE-epsilon4 effects on cognition. Neurobiol Aging.

[50] Stanimirovic DB, Friedman A (2012). Pathophysiology of the neurovascular unit: disease cause or consequence?. J Cereb Blood Flow Metab.

[51] Suarez-Calvet M, Morenas-Rodriguez E, Kleinberger G, Schlepckow K, Araque Caballero MA, Franzmeier N, Capell A, Fellerer K, Nuscher B, Eren E (2019). Early increase of CSF sTREM2 in Alzheimer's disease is associated with tau related-neurodegeneration but not with amyloid-beta pathology. Mol Neurodegener.

[52] Tan YL, Yuan Y, Tian L (2020). Microglial regional heterogeneity and its role in the brain. Mol Psychiatry.

[53] Tasaki S, Xu J, Avey DR, Johnson L, Petyuk VA, Dawe RJ, Bennett DA, Wang Y, Gaiteri C (2022). Inferring protein expression changes from mRNA in Alzheimer's dementia using deep neural networks. Nat Commun.

[54] Thal DR, Rub U, Orantes M, Braak H (2002). Phases of A beta-deposition in the human brain and its relevance for the development of AD. Neurology.

[55] Ulland TK, Song WM, Huang SC, Ulrich JD, Sergushichev A, Beatty WL, Loboda AA, Zhou Y, Cairns NJ, Kambal A (2017). TREM2 maintains microglial metabolic fitness in Alzheimer's disease. Cell.

[56] Ulrich JD, Ulland TK, Colonna M, Holtzman DM (2017). Elucidating the role of TREM2 in Alzheimer's disease. Neuron.

[57] Wang S, Mustafa M, Yuede CM, Salazar SV, Kong P, Long H, Ward M, Siddiqui O, Paul R, Gilfillan S (2020). Anti-human TREM2 induces microglia proliferation and reduces pathology in an Alzheimer's disease model. J Exp Med.

[58] Wang Y, Cella M, Mallinson K, Ulrich JD, Young KL, Robinette ML, Gilfillan S, Krishnan GM, Sudhakar S, Zinselmeyer BH (2015). TREM2 lipid sensing sustains the microglial response in an Alzheimer's disease model. Cell.

[59] Wu R, Li X, Xu P, Huang L, Cheng J, Huang X, Jiang J, Wu LJ, Tang Y (2017). TREM2 protects against cerebral ischemia/reperfusion injury. Mol Brain.

[60] Xu J, Farsad HL, Hou Y, Barclay K, Lopez BA, Yamada S, Saliu IO, Shi Y, Knight WC, Bateman RJ (2023). Human striatal glia differentially contribute to AD- and PD-specific neurodegeneration. Nature Aging.

[61] Zhao Y, Bhattacharjee S, Jones BM, Dua P, Alexandrov PN, Hill JM, Lukiw WJ (2013). Regulation of TREM2 expression by an NF-small ka, CyrillicB-sensitive miRNA-34a. NeuroReport.

[62] Zhao Y, Wu X, Li X, Jiang LL, Gui X, Liu Y, Sun Y, Zhu B, Pina-Crespo JC, Zhang M (2018). TREM2 is a receptor for beta-amyloid that mediates microglial function. Neuron.

[63] Zheng H, Jia L, Liu CC, Rong Z, Zhong L, Yang L, Chen XF, Fryer JD, Wang X, Zhang YW (2017). TREM2 promotes microglial survival by activating wnt/beta-catenin pathway. J Neurosci.

[64] Zhong L, Chen XF, Wang T, Wang Z, Liao C, Wang Z, Huang R, Wang D, Li X, Wu L (2017). Soluble TREM2 induces inflammatory responses and enhances microglial survival. J Exp Med.

[65] Zhong L, Xu Y, Zhuo R, Wang T, Wang K, Huang R, Wang D, Gao Y, Zhu Y, Sheng X (2019). Soluble TREM2 ameliorates pathological phenotypes by modulating microglial functions in an Alzheimer's disease model. Nat Commun.

