# Supplementary material for: TREM2 gene expression associations with Alzheimer’s disease neuropathology are region-specific: implications for cortical versus subcortical microglia
Source: Acta Neuropathol. 2023 Mar 25;145(6):733–47. doi: 10.1007/s00401-023-02564-2 (PMC10175463; doi:10.1007/s00401-023-02564-2)
Supplement: Supplementary file 1 — Supplementary file1 (DOCX 2517 KB) [file 401_2023_2564_MOESM1_ESM.docx]

**Supplemental Material**

**Supplemental Table 1. Main Effects of *TREM2* on Amyloid Outcome Measures Adjusting for Microglial Fraction**

| *Predictor (Tissue)* | *Outcome* | *B* | *SE* | *Pvalue* | *Covariate Inclusion* |
| --- | --- | --- | --- | --- | --- |
| dlPFC | Aβ_42_ | 0.446 | 0.090 | **5.07e-7** | Microglial Cell-Type Fraction |
| dlPFC | neuritic plaque | 0.183 | 0.044 | **1.13e-4** | Microglial Cell-Type Fraction |

**Supplemental Table 2. Regional Main Effects of *TREM2* on Amyloid and Tau**

| *TREM2 Region (Predictor)* | *Neuropathology Region* | *Neuropathology*  *Outcome* | *β* | *SE* | *Pvalue* |
| --- | --- | --- | --- | --- | --- |
| dlPFC, BA 46/9 | Superior frontal cortex, BA 6/8 | Aβ_1-42_ | 0.19 | 0.05 | **3.6e-4** |
| dlPFC, BA 46/9 | Midfrontal cortex, BA 46/9 | Aβ_1-42_ | 0.19 | 0.05 | **3.3e-4** |
| PCC, BA 23/31 | Superior frontal cortex, BA 6/8 | Aβ_1-42_ | 0.29 | 0.09 | **1.3e-3** |
| PCC, BA 23/31 | Midfrontal cortex, BA 46/9 | Aβ_1-42_ | 0.25 | 0.09 | **3.3e-3** |
|  | | | | | |
| dlPFC, BA 46/9 | Superior frontal cortex, BA 6/8 | p-tau | 0.15 | 0.06 | **0.01** |
| dlPFC, BA 46/9 | Midfrontal cortex, BA 46/9 | p-tau | 0.13 | 0.05 | **9.4e-3** |
| PCC, BA 23/31 | Superior frontal cortex, BA 6/8 | p-tau | 0.28 | 0.09 | **2.6e-3** |
| PCC, BA 23/31 | Midfrontal cortex, BA 46/9 | p-tau | 0.18 | 0.07 | **0.02** |

| *Predictor (Tissue)* | *Outcome* | *B* | *SE* | *Pvalue* | *Covariate Inclusion* |
| --- | --- | --- | --- | --- | --- |
| dlPFC | p-Tau, AT8 | 0.088 | 0.101 | 0.386 | Microglial Cell-Type Fraction |
| dlPFC | neurofibrillary tangles | 0.090 | 0.031 | **0.006** | Microglial Cell-Type Fraction |

**Supplemental Table 3.** **Main Effects of *TREM2* on Tau Outcome Measures Adjusting for Microglial Fraction**

**Supplemental Table 4. Main Effects of *TREM2* on Cerebrovascular Outcome Measures**

| ***TREM2* Predictor (Tissue)** | ***Outcome*** | ***β*** | ***SE*** | ***P-value*** | ***P.fdr*** |
| --- | --- | --- | --- | --- | --- |
| dlPFC | Cerebral Macro Infarcts | 0.159 | 0.102 | 0.119 | 0.199 |
| dlPFC | Cerebral Micro Infarcts | -0.110 | 0.091 | 0.228 | 0.342 |
| dlPFC | Arteriolosclerosis | -0.071 | 0.074 | 0.337 | 0.459 |
| dlPFC | Atherosclerosis | -0.176 | 0.075 | **0.019** | 0.061 |
| dlPFC | CAA | 0.143 | 0.074 | 0.052 | 0.131 |
| PCC | Cerebral Macro Infarcts | -0.028 | 0.167 | 0.866 | 0.896 |
| PCC | Cerebral Micro Infarcts | -0.040 | 0.148 | 0.788 | 0.876 |
| PCC | Arteriolosclerosis | 0.082 | 0.121 | 0.495 | 0.594 |
| PCC | Atherosclerosis | 0.005 | 0.124 | 0.967 | 0.967 |
| PCC | CAA | 0.203 | 0.120 | 0.090 | 0.168 |
| CN | Cerebral Macro Infarcts | 0.350 | 0.150 | **0.020** | 0.061 |
| CN | Cerebral Micro Infarcts | 0.175 | 0.130 | 0.180 | 0.284 |
| CN | Arteriolosclerosis | 0.256 | 0.105 | **0.015** | 0.061 |
| CN | Atherosclerosis | 0.179 | 0.107 | 0.095 | 0.168 |
| CN | CAA | -0.082 | 0.105 | 0.439 | 0.572 |

**Supplemental Table 5. Main Effects of *TREM2* on Cerebrovascular Outcome Measures Adjusting for Microglial Fraction**

| *Predictor (Tissue)* | *Outcome* | *β* | *SE* | *Pvalue* | *Covariate Inclusion* |
| --- | --- | --- | --- | --- | --- |
| dlPFC | Macro Infarcts | 0.070 | 0.220 | 0.752 | Microglial Cell-Type Fraction |
| dlPFC | Micro Infarcts | -0.228 | 0.196 | 0.245 | Microglial Cell-Type Fraction |
| dlPFC | Arteriolosclerosis | -0.210 | 0.150 | 0.162 | Microglial Cell-Type Fraction |
| dlPFC | Atherosclerosis | -0.165 | 0.158 | 0.294 | Microglial Cell-Type Fraction |
| dlPFC | CAA | 0.245 | 0.154 | 0.112 | Microglial Cell-Type Fraction |

**Supplemental Table 6 Cohort Characteristics Microglia Subset**

| **Characteristic** | **Cortical** | **Caudate** |
| --- | --- | --- |
| N | 156 | 104 |
| AD pathological diagnosis, no. (%) | 89 (57) | 57 (55) |
| AD clinical diagnosis, no. (%) | 53 (34) | 33 (32) |
| Other dementia clinical diagnosis, no (%) | 3 (2) | 1 (<1) |
| MCI clinical diagnosis, no (%) | 50 (32) | 36 (35) |
| No cognitive impairment, no (%) | 50 (32) | 34 (33) |
| APOE4 Carrier, no. (%) | 37 (24) | 24 (23) |
| Male, no. (%) | 58 (37) | 39 (38) |
| White, no. (%) | 156 (100) | 104 (100) |
| Age at death (years) | 89.4 +/- 5.6 | 89.2 +/- 5.6 |
| Education (years) | 15.0 +/- 2.7 | 14.6 +/- 2.4 |
| Global cognition | -0.8 +/- 1.0 | -0.7 +/- 0.9 |
| PMI (hours) | 6.8 +/- 3.3 | 6.8 +/- 3.2 |

**Supplemental Table 7. *TREM2* * Neuropathology Interactions on Activated Microglial Density (PAM) in the Caudate**

| Interaction Term (neuropathology) | β | SE | P-value |
| --- | --- | --- | --- |
| Cerebral Macro Infarcts | 1.720 | 2.824 | 0.544 |
| Cerebral Micro Infarcts | -0.013 | 2.517 | 0.996 |
| Arteriolosclerosis | -1.228 | 1.09 | 0.262 |
| Atherosclerosis | 1.222 | 1.105 | 0.272 |
| CAA | 0.789 | 1.246 | 0.528 |
| Aβ_1-42_ | 0.165 | 0.808 | 0.838 |
| neuritic plaque | -0.853 | 1.942 | 0.661 |
| p-Tau, AT8 | 0.193 | 0.933 | 0.837 |
| neurofibrillary tangles | -2.107 | 3.014 | 0.486 |

**Supplemental Table 8. Main Effects of Microglial Activation Markers on Amyloid**

| *Gene (Predictor)* | *Brain Region* | | *Neuropathology Outcome* | *β* | *SE* | *Pvalue* |
| --- | --- | --- | --- | --- | --- | --- |
| CD44 | dlPFC | | Aβ_1-42_ | 0.129 | 0.030 | **1.7e-5** |
| CD45 | dlPFC | | Aβ_1-42_ | 0.094 | 0.048 | **0.050** |
| CD11B | dlPFC | | Aβ_1-42_ | 0.122 | 0.057 | **0.034** |
| SPP1 | dlPFC | | Aβ_1-42_ | 0.2369 | 0.038 | **4.4e-10** |
| LGALS3 | dlPFC | | Aβ_1-42_ | -0.055 | 0.057 | 0.331 |
|  | | | | | | |
| CD44 | PCC | | Aβ_1-42_ | 0.11 | 0.041 | **0.007** |
| CD45 | PCC | | Aβ_1-42_ | 0.089 | 0.073 | 0.224 |
| CD11B | PCC | | Aβ_1-42_ | 0.154 | 0.094 | 0.101 |
| SPP1 | PCC | | Aβ_1-42_ | 0.145 | 0.041 | **4.6e-4** |
| LGALS3 | PCC | | Aβ_1-42_ | -0.118 | 0.079 | 0.136 |
|  | | | | | | |
| CD44 | | CN | Aβ_1-42_ | -0.04 | 0.03 | 0.22 |
| CD45 | | CN | Aβ_1-42_ | -0.016 | 0.064 | 0.805 |
| CD11B | | CN | Aβ_1-42_ | 0.052 | 0.090 | 0.567 |
| SPP1 | | CN | Aβ_1-42_ | 0.103 | 0.058 | 0.077 |
| LGALS3 | | CN | Aβ_1-42_ | -0.124 | 0.082 | 0.133 |

**Supplemental Fig. 1 *TREM2* mRNA expression does not differ by *APOE*-ε4 carrier status.** **(a)** Dorsolateral prefrontal cortex (dlPFC) *TREM2* mRNA levels across *APOE* status. **(b)** Posterior cingulate cortex (PCC) *TREM2* mRNA levels across *APOE* status. **(c)** Head of caudate nucleus (CN) *TREM2* mRNA levels across *APOE* status. P-values represent statistical comparison of means by a student’s t-test.


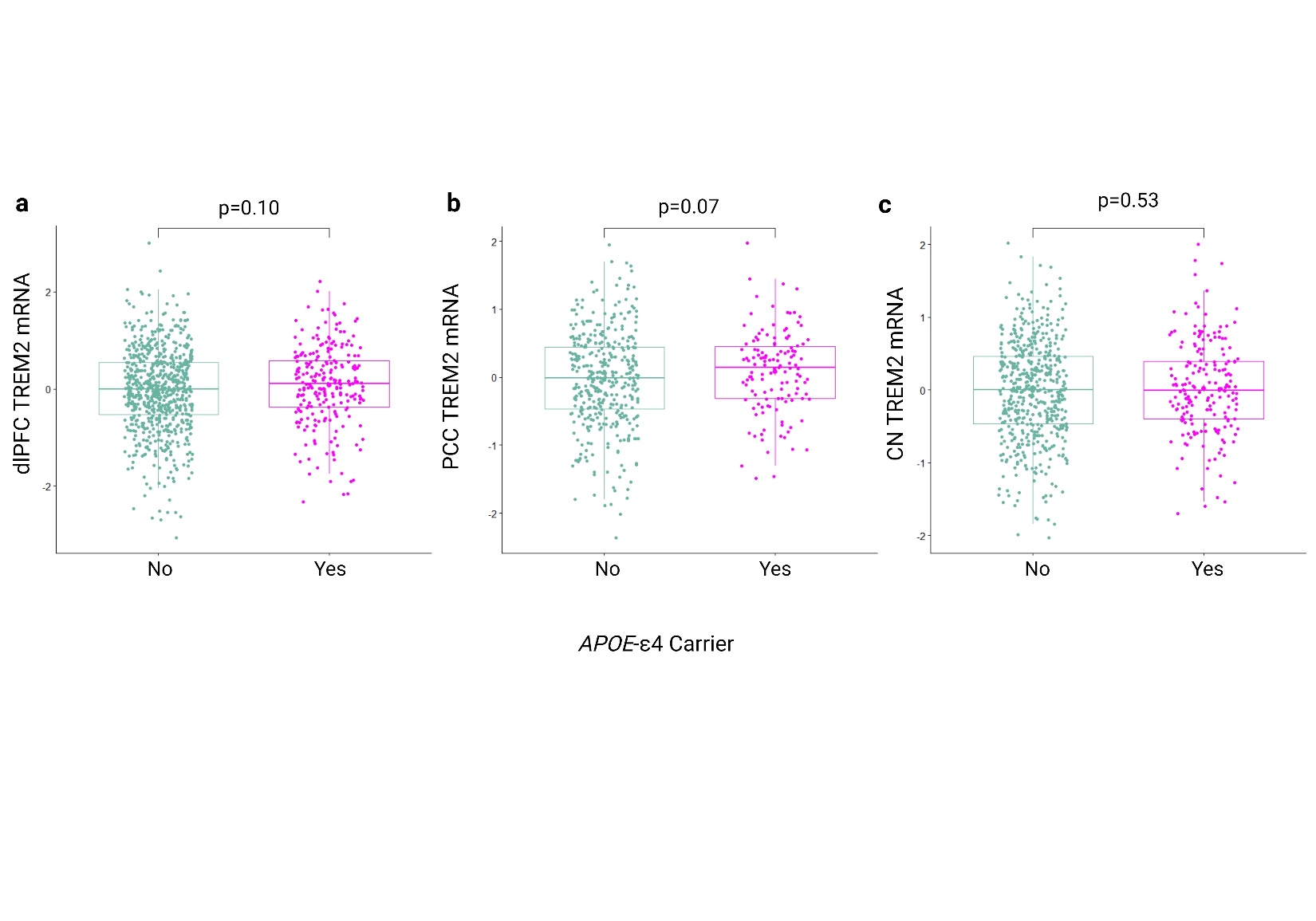


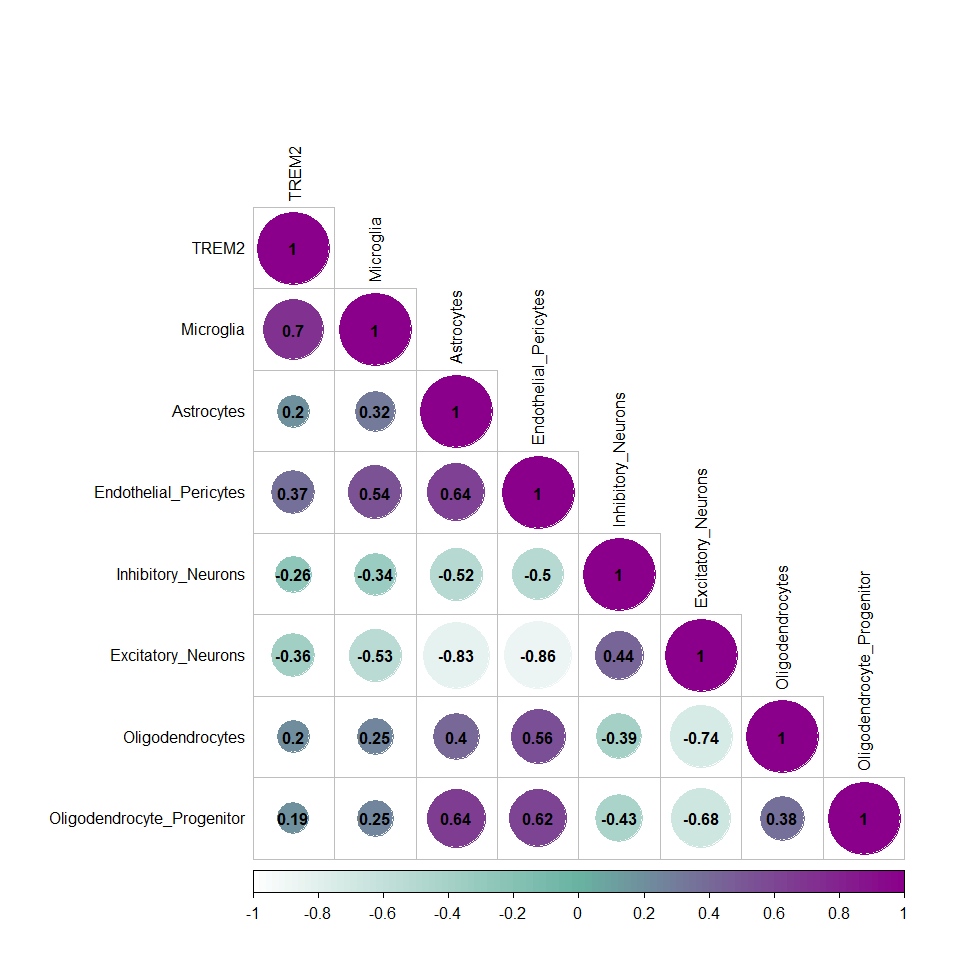


**Supplemental Fig. 2** ***TREM2* correlations with cell-type fraction.** *TREM2* mRNA is significantly correlated with microglial cell-type fraction. Representative data shows *TREM2* from the dorsolateral prefrontal cortex (dlPFC) is significantly correlated with microglial cell-type fraction above other cell types examined. A Pearson’s correlation coefficient (r) is displayed for each comparison.

**Supplemental Fig. 3** ***TREM2* associations with amyloid stratified by diagnosis**. Positive associations between cortical*TREM2* mRNA and amyloid neuropathology are largely driven by AD cases while caudate *TREM2* mRNA is negatively associated with amyloid neuropathology in AD cases but not controls. **(a-c)** Regional *TREM2* mRNA levels by beta-amyloid (Aβ_1-42_) burden as measured by immunohistochemistry. **(d-f)** Regional *TREM2* mRNA levels by neuritic plaque burden as measured by silver stain. Shown are unadjusted scatter plots. Final summary clinical diagnosis includes: no cognitive impairment and mild cognitive impairment (AD diagnosis negative) and Alzheimer’s disease dementia (AD diagnosis positive). Dorsolateral prefrontal cortex (dlPFC), caudate nucleus (CN), and posterior cingulate cortex (PCC).


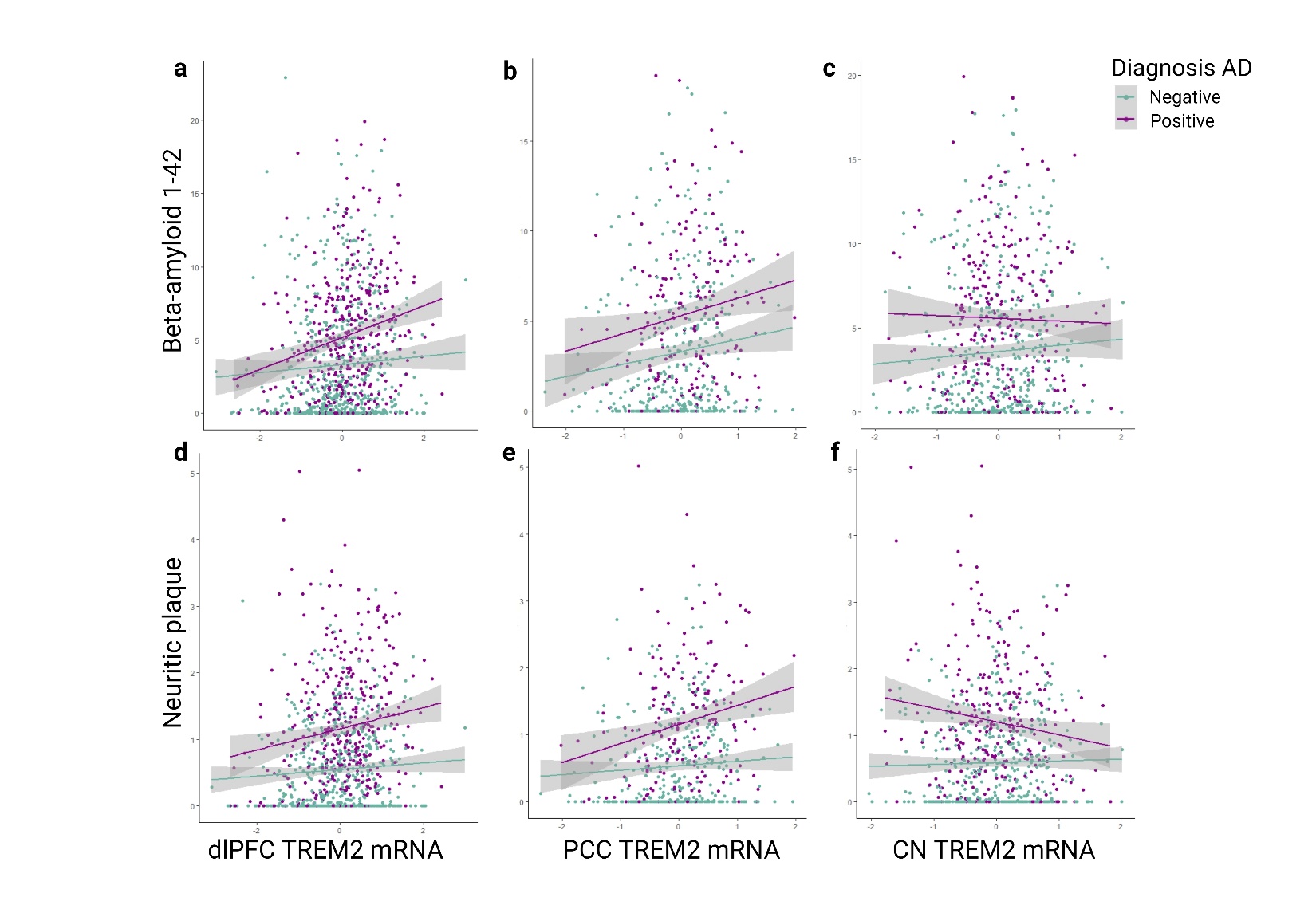


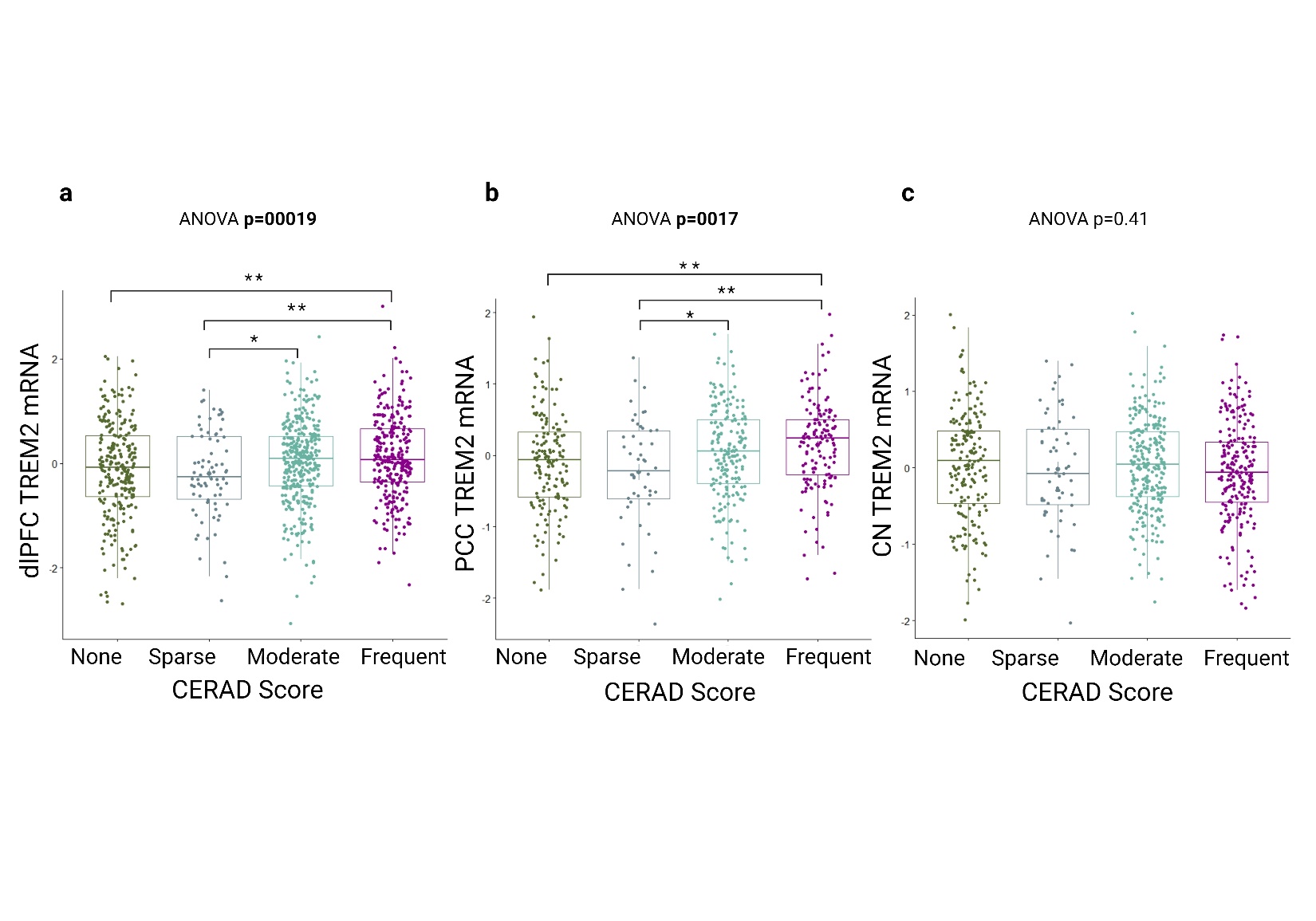


**Supplemental Fig. 4** ***TREM2* levels across CERAD scores**. Cortical but not caudate levels of *TREM2* mRNA at autopsy differ across pathological severity. Participants with higher amyloid burden (CERAD “moderate” or “frequent”) tend to have higher levels of cortical *TREM2* mRNA at autopsy compared to participants with lower amyloid burden (CERAD “sparse” or “none”). **(a)** Dorsolateral prefrontal cortex (dlPFC) *TREM2* mRNA levels across CERAD scores increase with increasing amyloid burden. **(b)** Posterior cingulate cortex (PCC) *TREM2* mRNA levels across CERAD scores increase with increasing amyloid burden. **(c)** *TREM2* mRNA levels measured from the caudate nucleus (CN) do not differ across pathological severity. Significance as determined by individual comparison of means (student’s t-test) is denoted as follows: one asterisk; P≤0.05, two asterisks; P≤0.01, and three asterisks; P≤0.001. Consortium to Establish a Registry for Alzheimer’s disease (CERAD) protocol for neuritic amyloid plaque density scores.


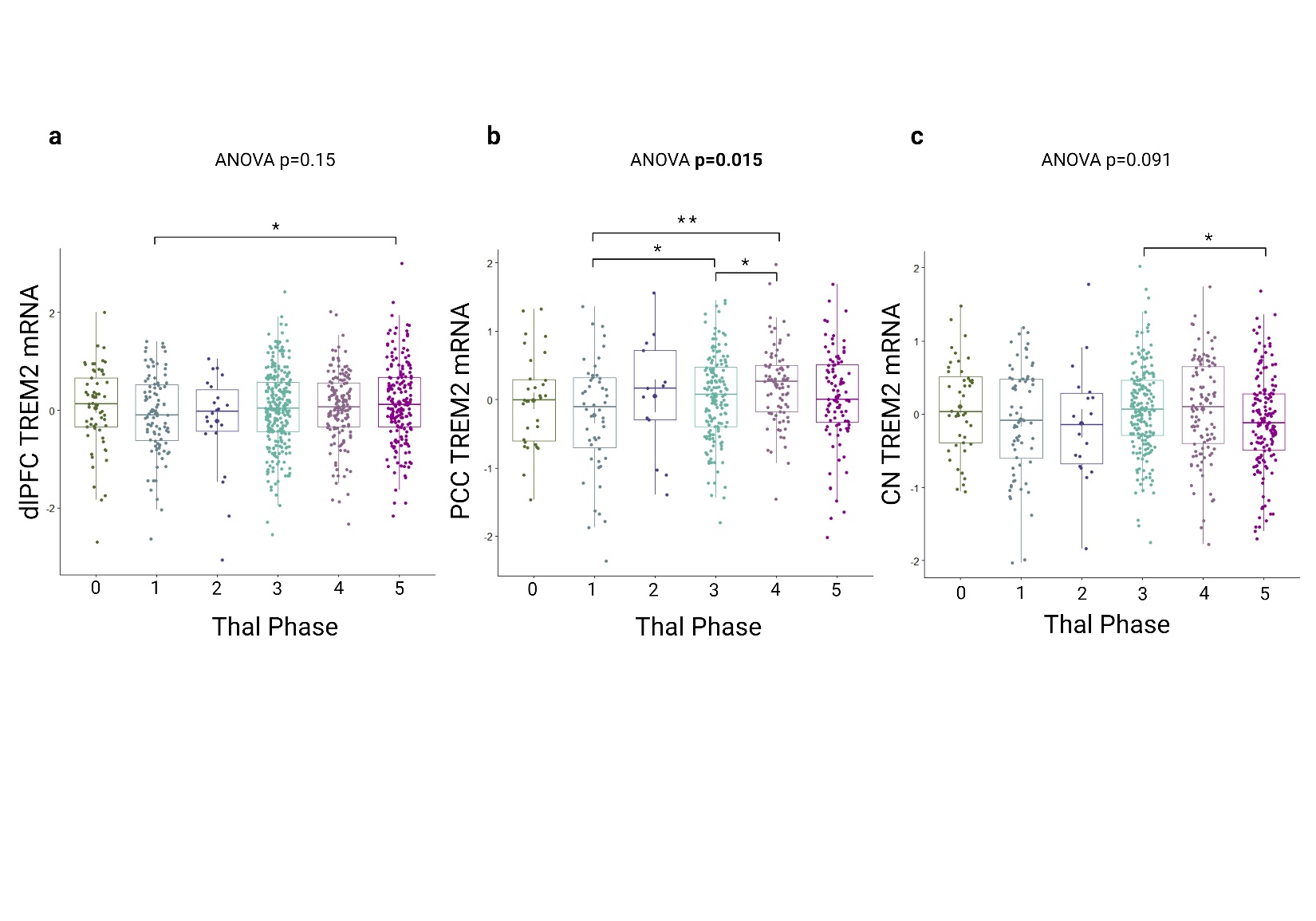


**Supplemental Fig. 5** ***TREM2* levels across Thal Phases**. Posterior cingulate cortex (PCC) but not dorsolateral prefrontal cortex (dlPFC) or caudate nucleus (CN) levels of *TREM2* mRNA at autopsy differ across Thal phases of progressive amyloid deposition [0; none, 1; neocortex only, 2; neocortex and hippocampus, 3; neocortex (with or without the hippocampus) and basal ganglia, 4; neocortex (with or without the hippocampus), basal ganglia, and substantia nigra, 5; neocortex (with or without the hippocampus), basal ganglia (with or without the substantia nigra), and cerebellum] according to an Analysis of Variance (ANOVA). **(a)** Regional results for dlPFC show no significant differences by ANOVA in *TREM2* mRNA expression across Thal stages. **(b)** Regional results for PCC suggest subtle changes in *TREM2* mRNA expression across Thal phases 1-4. **(c)** Regional results for CN show no significant differences by ANOVA in *TREM2* mRNA expression across Thal stages. Significance as determined by individual comparison of means (student’s t-test) is denoted as follows: one asterisk; P≤0.05, two asterisks; P≤0.01, and three asterisks; P≤0.001.

**Supplemental Fig. 6** **Higher caudate *TREM2* is associated with lower phosphorylated tau in amyloid positive individuals.** Amyloid status based on Consortium to Establish a Registry for Alzheimer’s Disease (CERAD) scoring of neuritic plaques. Amyloid Positive: CERAD probable or definite. Amyloid Negative: CERAD possible or no AD.

**
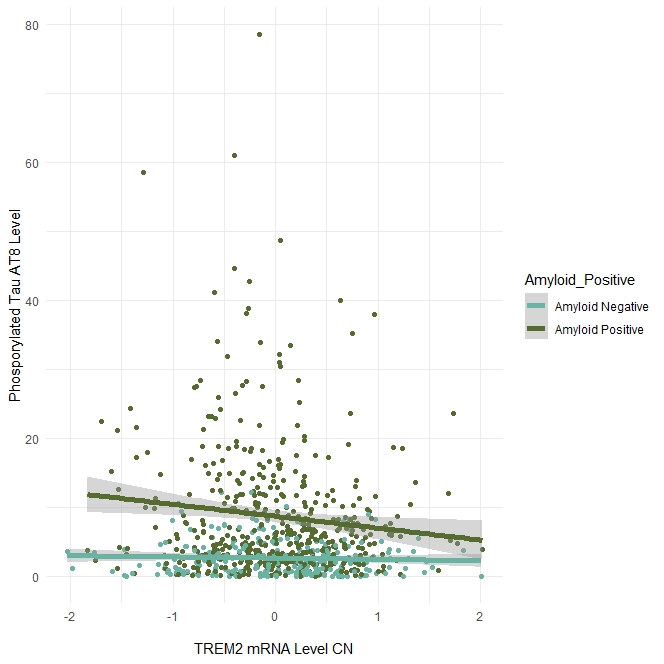
**

**Supplemental Fig. 7** ***TREM2* associations with tau stratified by diagnosis**. Positive associations between cortical*TREM2* mRNA and tau neuropathology are largely driven by AD cases while caudate *TREM2* mRNA is negatively associated with tau neuropathology in AD cases only. **(a-c)** Regional *TREM2* mRNA levels by phosphorylated tau (AT8 epitope, Ser202/Thr305) burden as measured by immunohistochemistry. **(d-f)** Regional *TREM2* mRNA levels by neurofibrillary burden as measured by silver stain. Shown are unadjusted scatter plots. Final summary clinical diagnosis includes: no cognitive impairment and mild cognitive impairment (AD diagnosis negative) and Alzheimer’s disease dementia (AD diagnosis positive). Dorsolateral prefrontal cortex (dlPFC), caudate nucleus (CN), and posterior cingulate cortex (PCC).


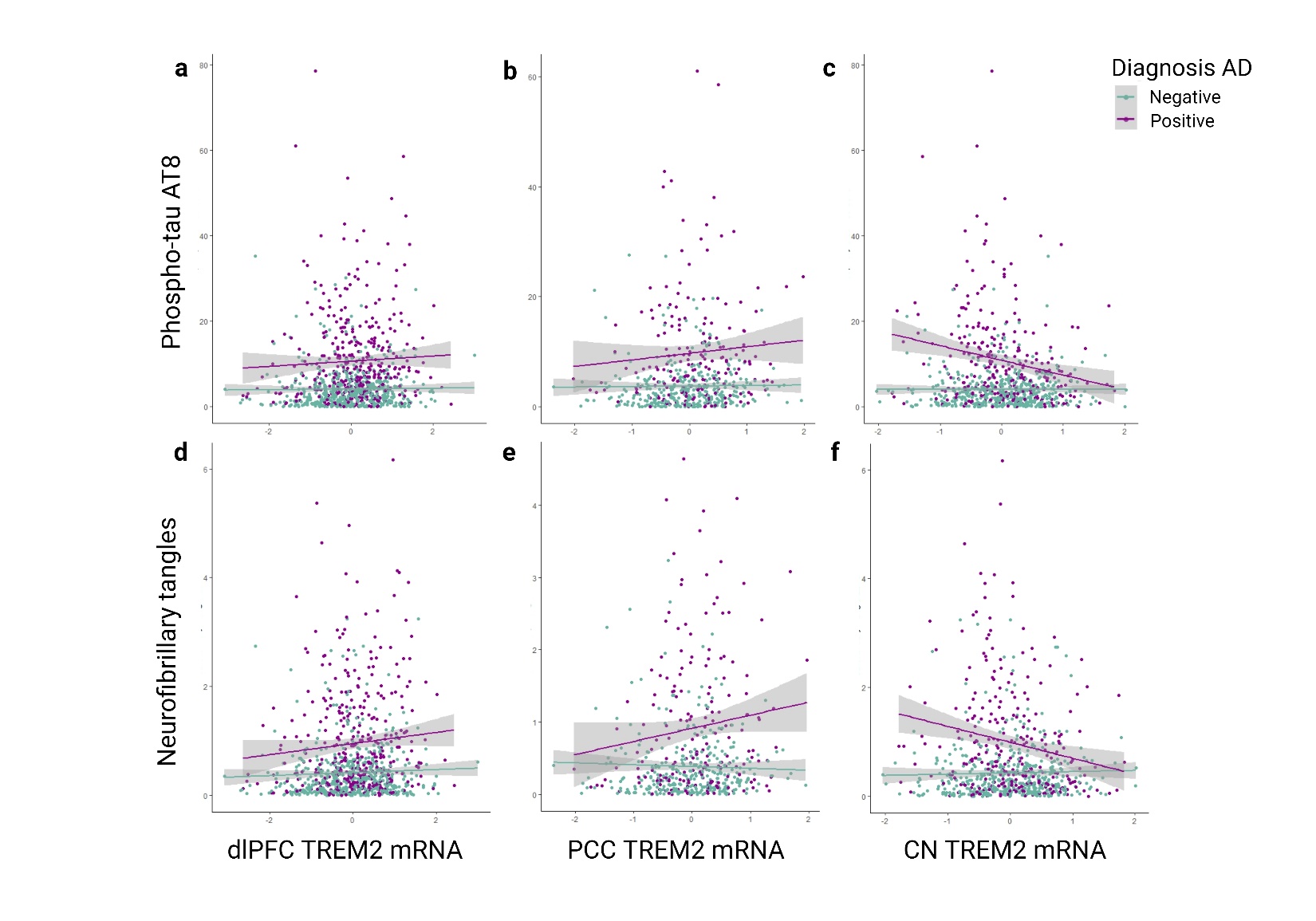


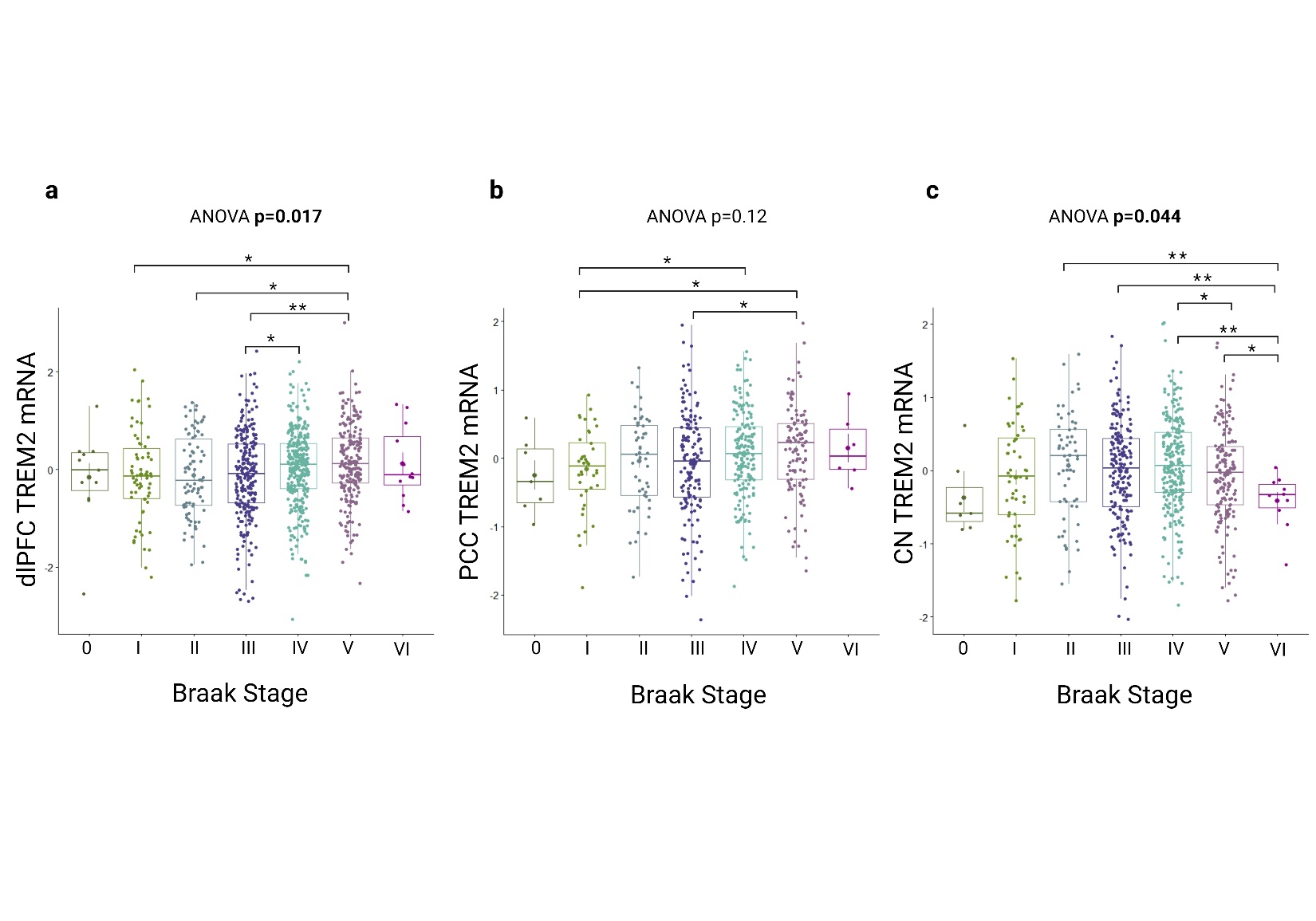


**Supplemental Fig. 8** ***TREM2* levels across Braak Stages**. Dorsolateral prefrontal cortex (dlPFC) and caudate nucleus (CN) but not posterior cingulate cortex (PCC) levels of *TREM2* mRNA at autopsy differ across Braak stages of neurofibrillary tangle (NFT) pathology according to an Analysis of Variance (ANOVA). Stage 0; no tangles, stages I and II indicate NFT pathology confined to the entorhinal region, stages III and IV indicate NFT pathology in limbic regions (i.e., hippocampus), and stages V and VI indicate moderate to severe NFT pathology in neocortex. **(a)** Regional results for dlPFC show significant increases in *TREM2* mRNA expression across multiple Braak stages. **(b)** Regional results for PCC show no significant differences by ANOVA in *TREM2* mRNA expression across Braak staging. **(c)** Regional results for CN show decreases in *TREM2* mRNA expression across Braak stages 2-6. Significance as determined by individual comparison of means (student’s t-test) is denoted as follows: one asterisk; P≤0.05, two asterisks; P≤0.01, and three asterisks; P≤0.001.

**Supplemental Fig. 9** ***TREM2* correlations with microglial density components stratified by diagnosis. (a)** Caudate but not cortical *TREM2* mRNA is positively correlated with total microglial density in the mfCx in control participants with a negative AD diagnosis. **(b)** Caudate but not cortical *TREM2* mRNA is significantly correlated with the proportion of activated microglial density (PAM) cis-regionally in AD cases but not controls. Midfrontal cortex (mfCx); ventral medial caudate (vmCaudate). A Pearson’s correlation coefficient (r) is displayed for each comparison. An asterisk denotes significance set to an *a priori* threshold of p<0.05. Final summary clinical diagnosis includes: no cognitive impairment and mild cognitive impairment (AD diagnosis negative) and Alzheimer’s disease dementia (AD diagnosis positive).


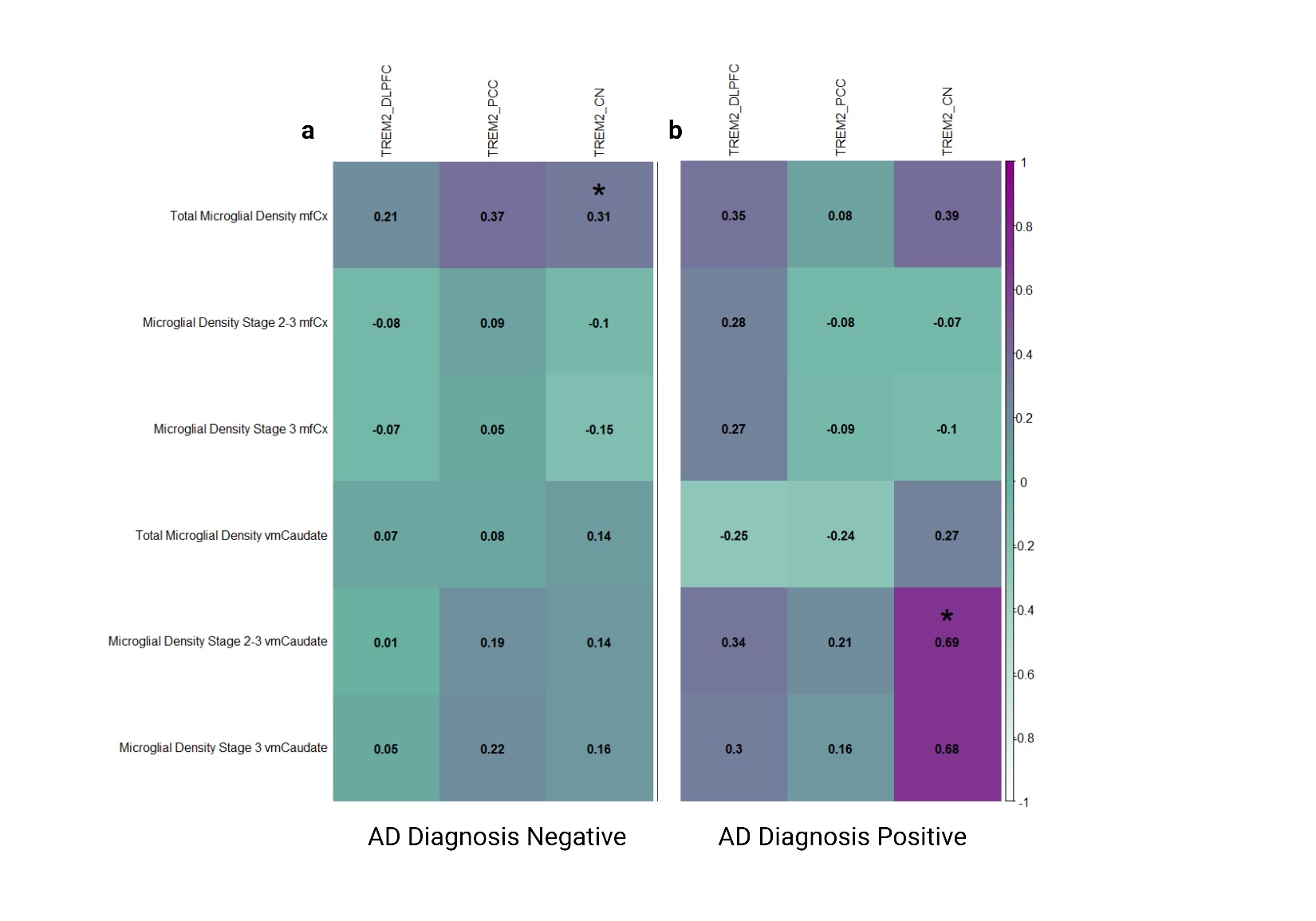


**Supplemental Fig. 10** ***TREM2* association with the proportion of activated microglia density (PAM) stratified by diagnosis.** Caudate *TREM2* mRNA levels by the proportion of activated microglial density (PAM) stages 2-3. Final summary clinical diagnosis includes: no cognitive impairment and mild cognitive impairment (AD diagnosis negative) and Alzheimer’s disease dementia (AD diagnosis positive). Shown is the unadjusted scatter plot.


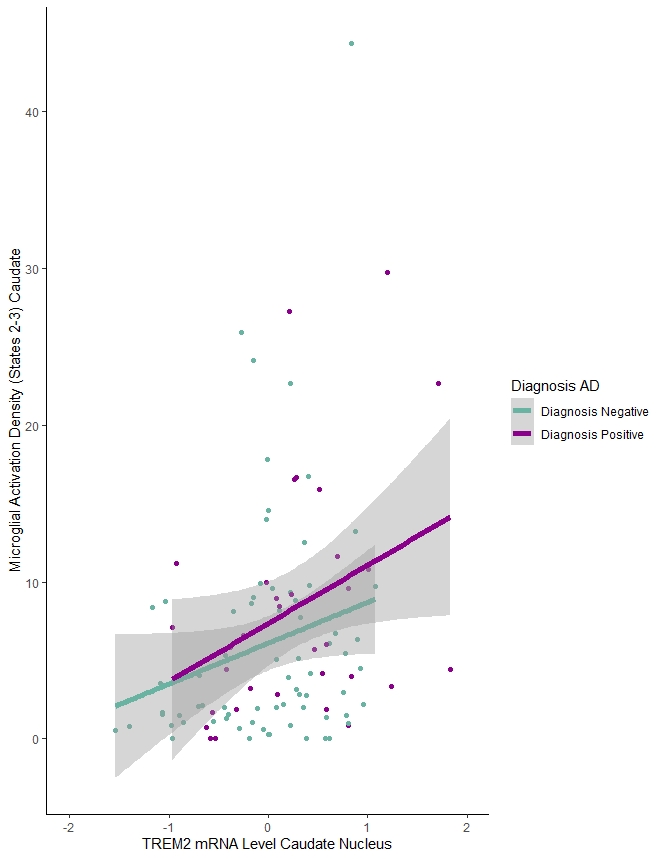


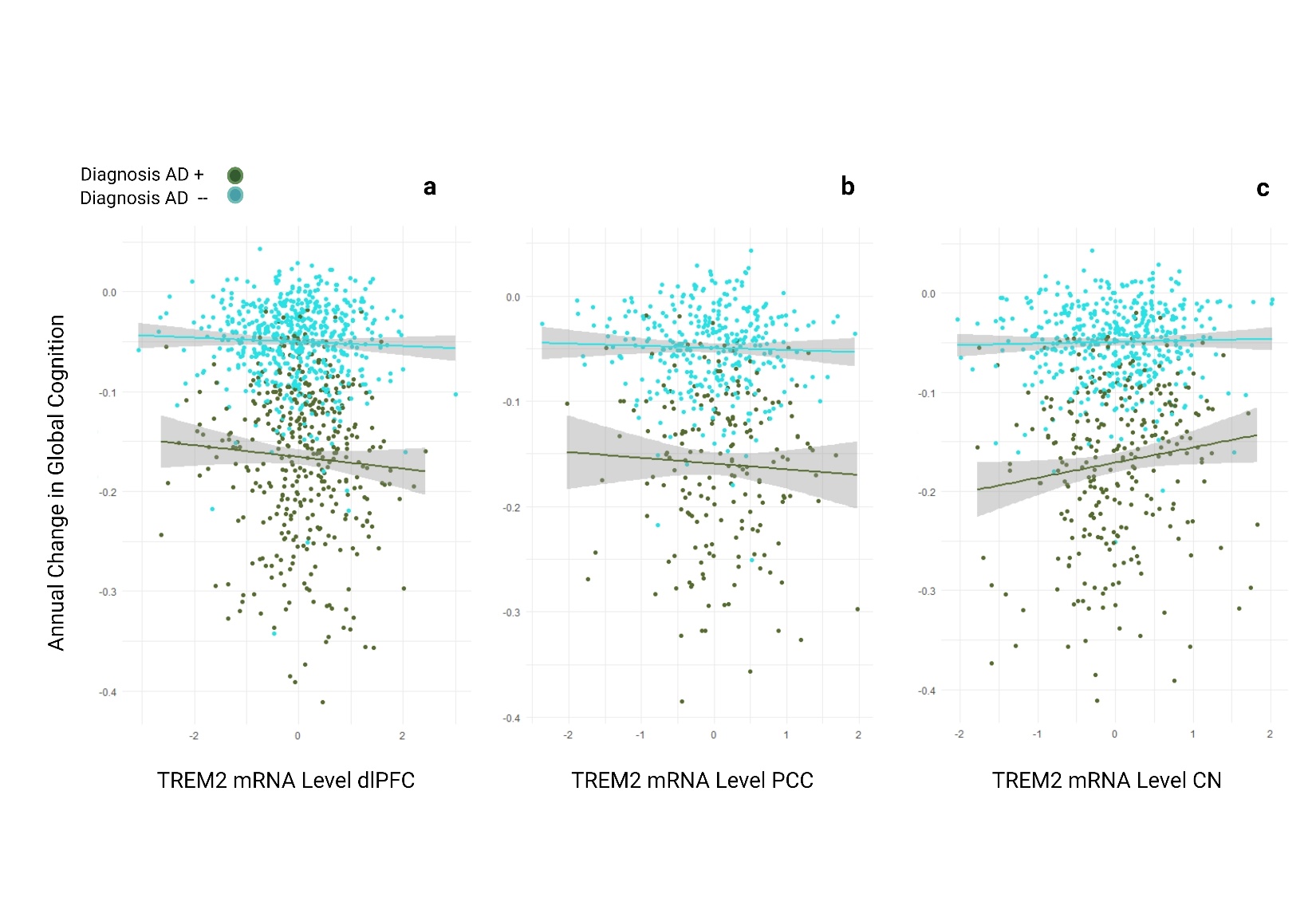


**Supplemental Fig. 11** ***TREM2* interactions with AD diagnosis on cognition**. *TREM2* mRNA levels in AD participants differentially relate to the annual change in global cognition depending on measurement from caudate or cortical brain regions. **(a)** High dorsolateral prefrontal cortex (dlPFC) *TREM2* mRNA levels relate to a more rapid decline in annual change in global cognition. **(b)** High posterior cingulate cortex (PCC) *TREM2* mRNA levels relate to a more rapid decline in global cognition and **(c)** high head of caudate nucleus (CN) *TREM2* mRNA levels related to a slower decline in global cognition in AD participants only.

**
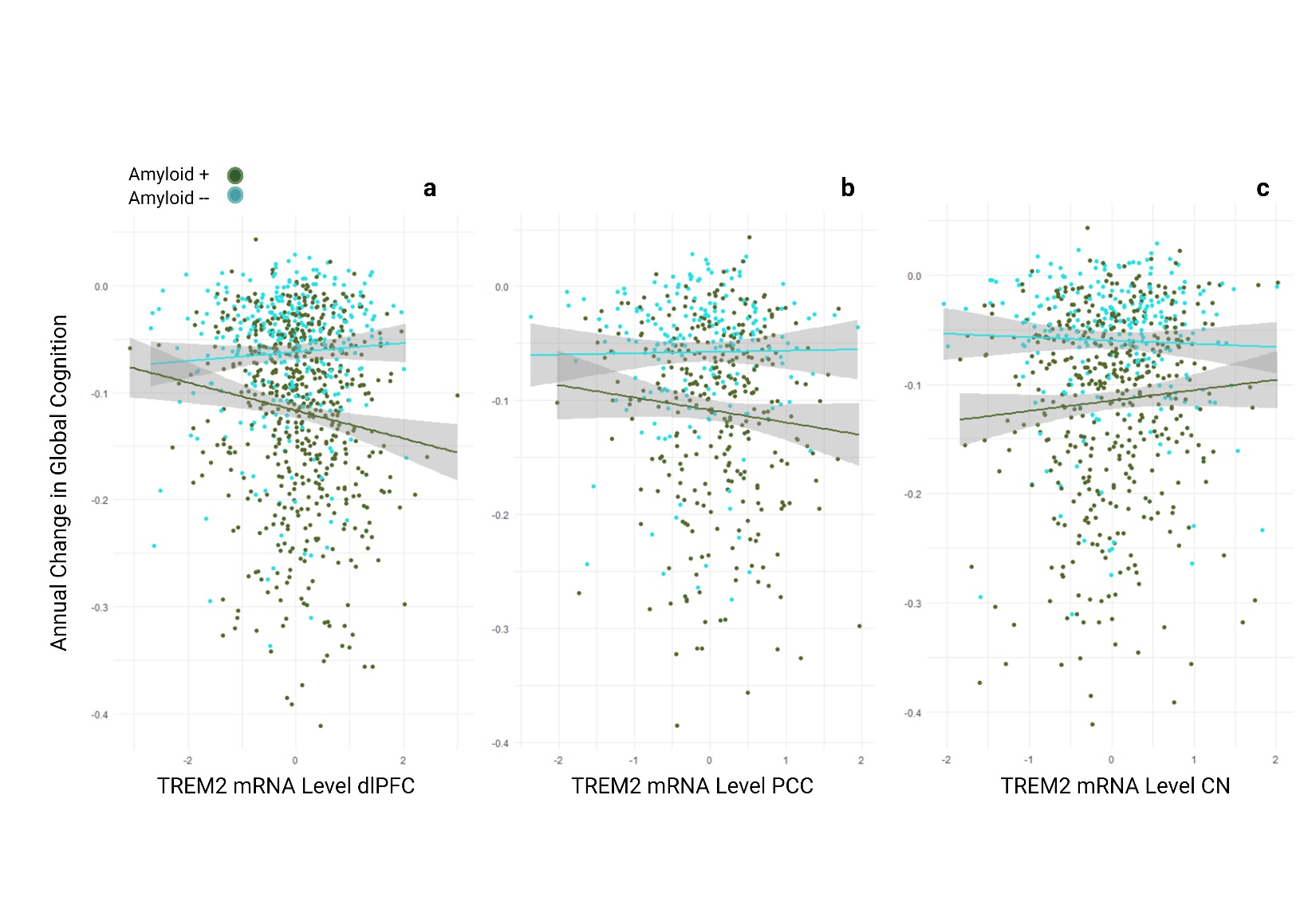
**

**Supplemental Fig. 12** ***TREM2* interactions with amyloid status on cognition**. High *TREM2* cortical but not caudate mRNA at autopsy predicts retrospective cognitive decline in amyloid positive individuals. **(a)** Dorsolateral prefrontal cortex (dlPFC) *TREM2* mRNA levels by annual change in global cognition. **(b)** Posterior cingulate cortex (PCC) *TREM2* mRNA levels and **(c)** head of caudate nucleus (CN) *TREM2* mRNA levels.


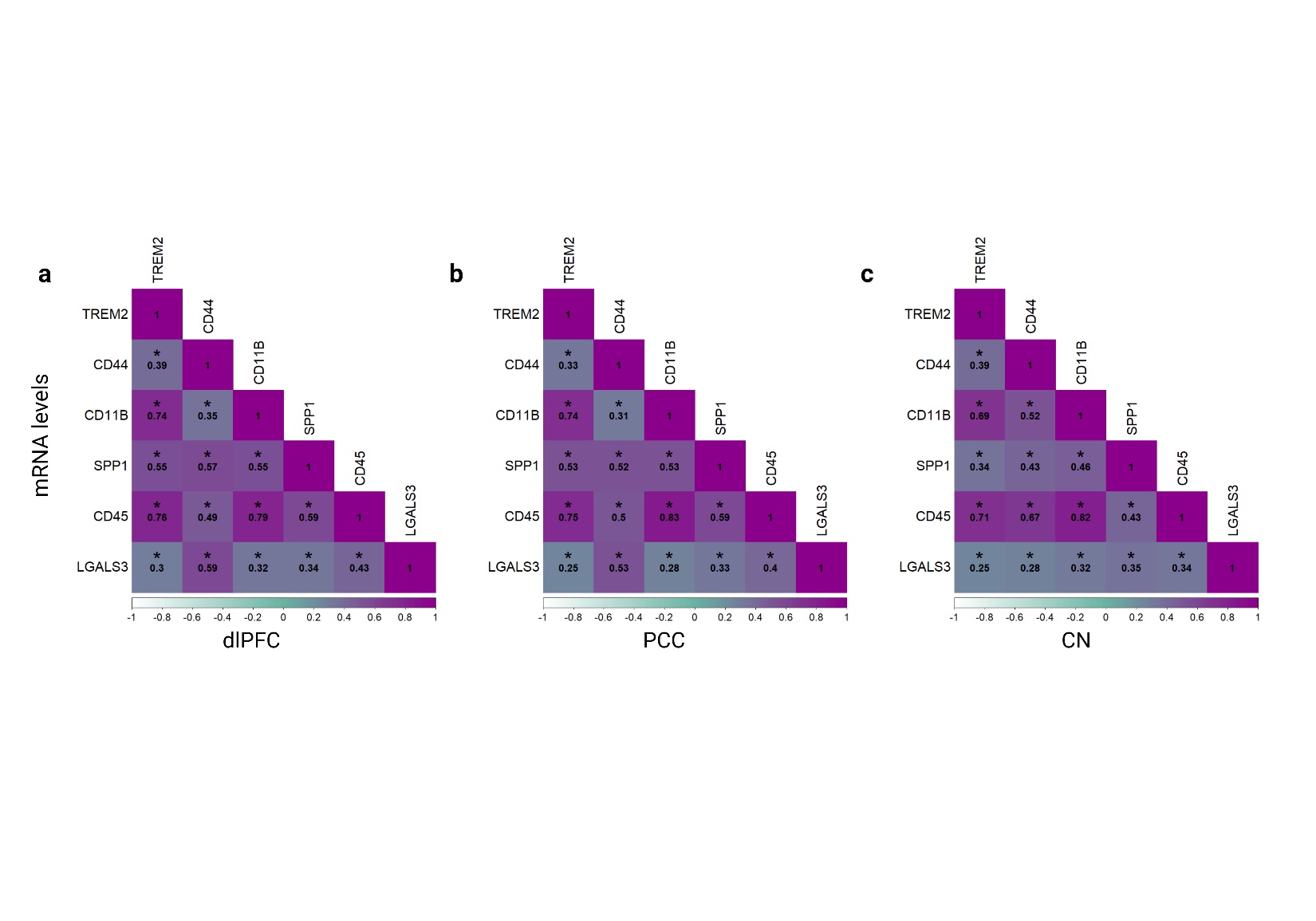


**Supplemental Fig. 13** **Regional** ***TREM2* correlations with microglial activation markers.** *TREM2* mRNA is positively correlated with microglial activation transcripts across brain regions. A Pearson’s correlation coefficient (r) is displayed for each comparison. **(a)** Transcript measurements from dorsolateral prefrontal cortex (dlPFC), **(b)** caudate nucleus (CN), and **(c)** posterior cingulate cortex (PCC).
